# Supplementary material for: Using the app “Injurymap” to provide exercise rehabilitation for people with acute lateral ankle sprains seen at the Hospital Emergency Department–A mixed-method pilot study
Source: PLOS Digit Health. 2023 May 15;2(5):e0000221. doi: 10.1371/journal.pdig.0000221 (PMC10184914; doi:10.1371/journal.pdig.0000221)
Supplement: S1 Table — (DOCX) [file pdig.0000221.s001.docx]

**S1 Table: Good Reporting of A Mixed Methods Study (GRAMMS) checklist**

| **Item** | **Reported** |
| --- | --- |
| Describe the justification for using a mixed methods approach to the research question | Purpose statement, page 3. |
| Describe the design in terms of the purpose, priority and sequence of methods | Study design, page 4 + Figure 1 |
| Describe each method in terms of sampling, data collection and analysis | Outcomes, pages 6-8 |
| Describe where integration has occurred, how it has occurred and who has participated in it | Study design, page 4 + Figure 1 + Table 5. |
| Describe any limitation of one method associated with the present of the other method | Strengths and limitations, pages 13-14. |
| Describe any insights gained from mixing or integrating methods | Discussion, pages 11-14 |

Reference: O'Cathain A, Murphy E, Nicholl J. The quality of mixed methods studies in health services research. J Health Serv Res Policy. 2008;13(2):92-98.
