# Supplementary material for: Using the app “Injurymap” to provide exercise rehabilitation for people with acute lateral ankle sprains seen at the Hospital Emergency Department–A mixed-method pilot study
Source: PLOS Digit Health. 2023 May 15;2(5):e0000221. doi: 10.1371/journal.pdig.0000221 (PMC10184914; doi:10.1371/journal.pdig.0000221)
Supplement: S2 Table — (DOCX) [file pdig.0000221.s002.docx]

S2 Table: Exercise program.

Exercises in phase 1 of the program

| Phase 1 | | | |
| --- | --- | --- | --- |
| Mobility | **Stability/balance** | **Strength** | **Stretch** |
| 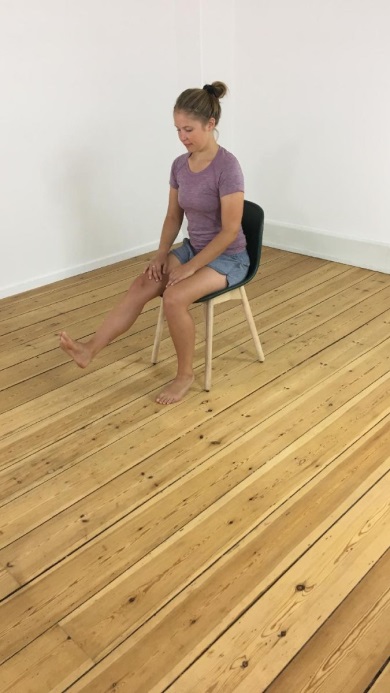Mob. 1.1: Ankle bendings. | **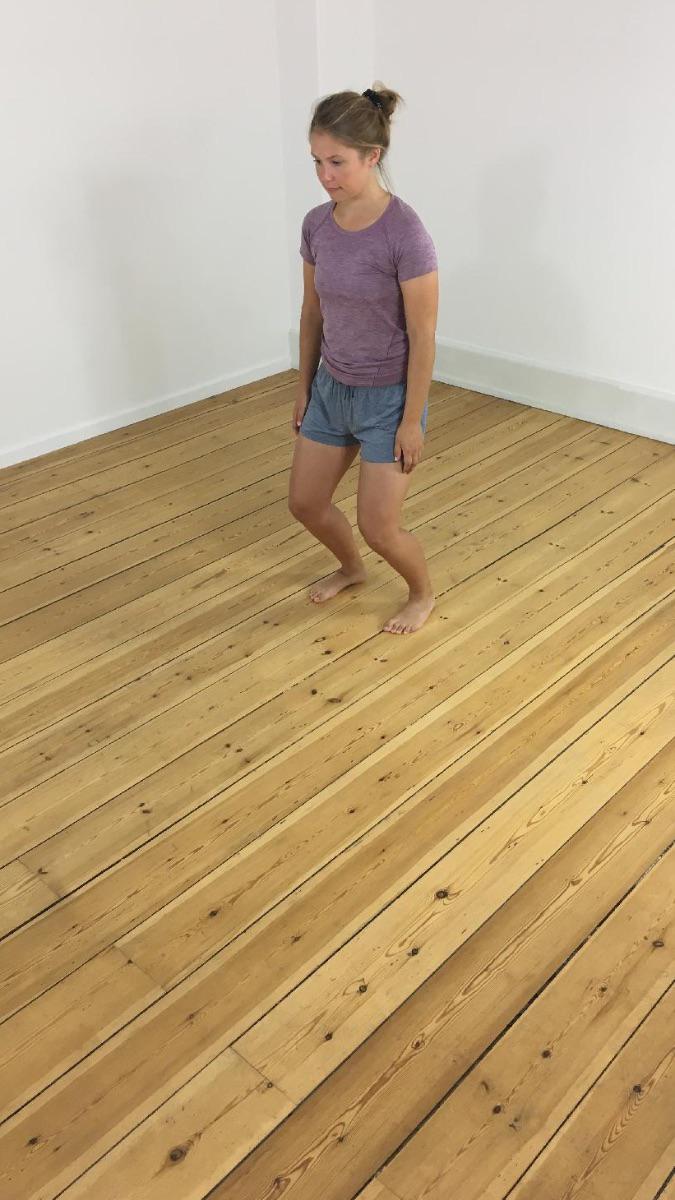Stab. 1.1 Ankle Balance** | **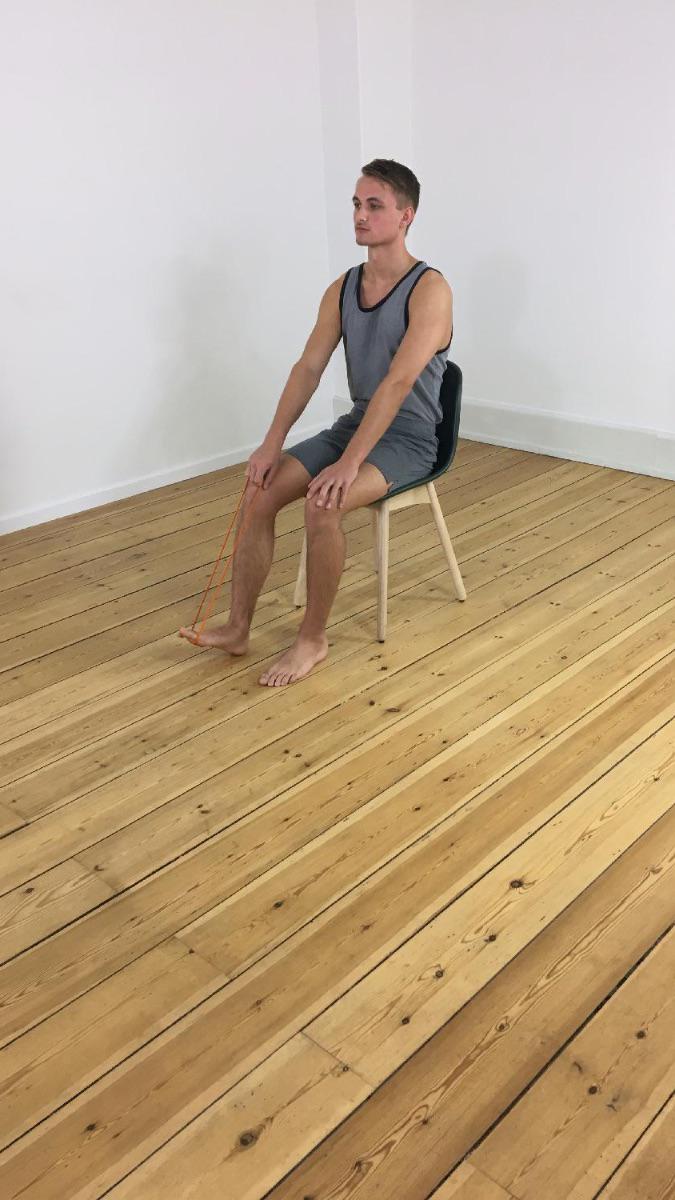Strength 1.1: Sitting ankle extensions I** | **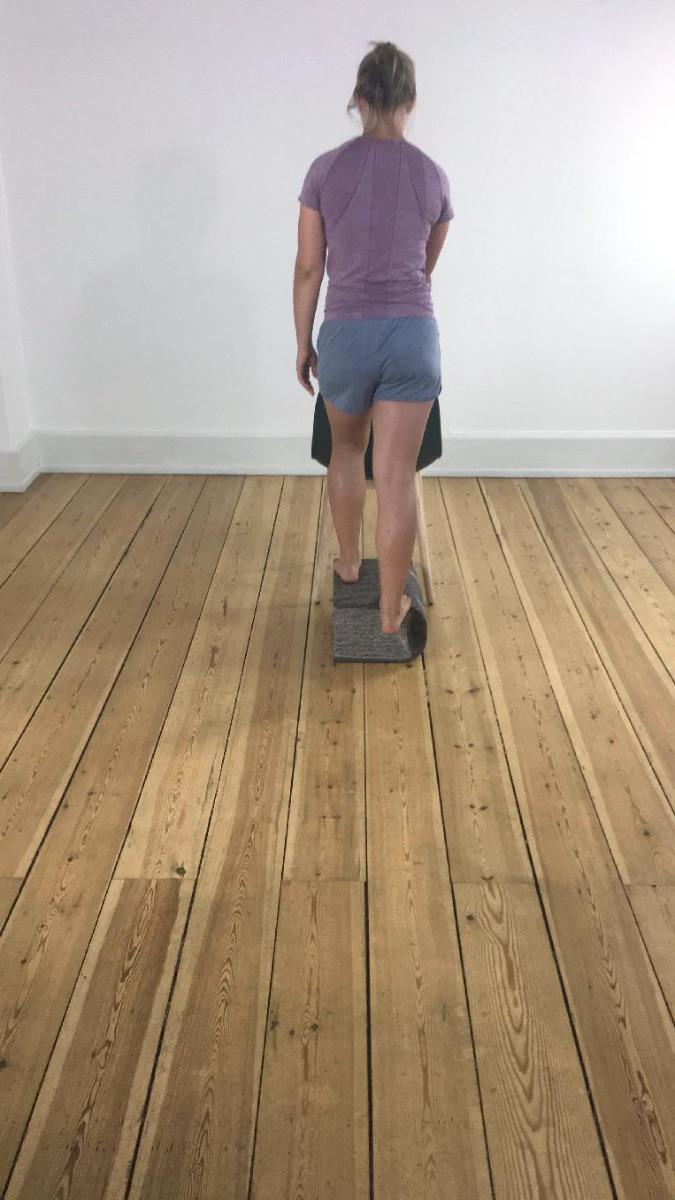Stretch 1.1: Straight leg calf stretch** |
| Sit on a chair. Lift the one foot from the floor. Bend the foot upwards and downwards as far as possible. If the foot is swelled the exercise can be done lying on the floor with the leg and foot raised above the heart.  10 reps each foot. | **Level I**  Stand with shoulder width stance and the feet pointing forward. Bend your knees as far beyond the toes as possible so that the ankles bend to their maximum. You should feel it tightens in the back of the heel and clamps in the front, but it must not be painful. Keep the tempo slow and controlled.  10 reps.  **Level II:** Perform the exercise while looking from side to side. | **Level I:** Sit on a chair with the knees bend. Place the rubber band under one foot just behind the toes. Hold the other end of the rubber band with one hand and tighten it.  Extend the foot downwards while using the rubber band to manage resistance. Return slowly. Move as far as possible.  The rubber band needs to be so tight that it is only possible to perform  10 reps x 3 sets.  **Level II:** Perform the exercise with the knees extended.  15 reps x 3 sets. | Stand on a stair step with one foot only touching the step with the forefoot and the heel free from the edge.  Lower the heel downwards with the knee extended until you feel a stretch in the calf muscles. Put as much weight on the leg as possible without provoking pain.  Keep stretch position 30 sec. x 3 reps. |
| Mob. 1.2: Ankle side tilts  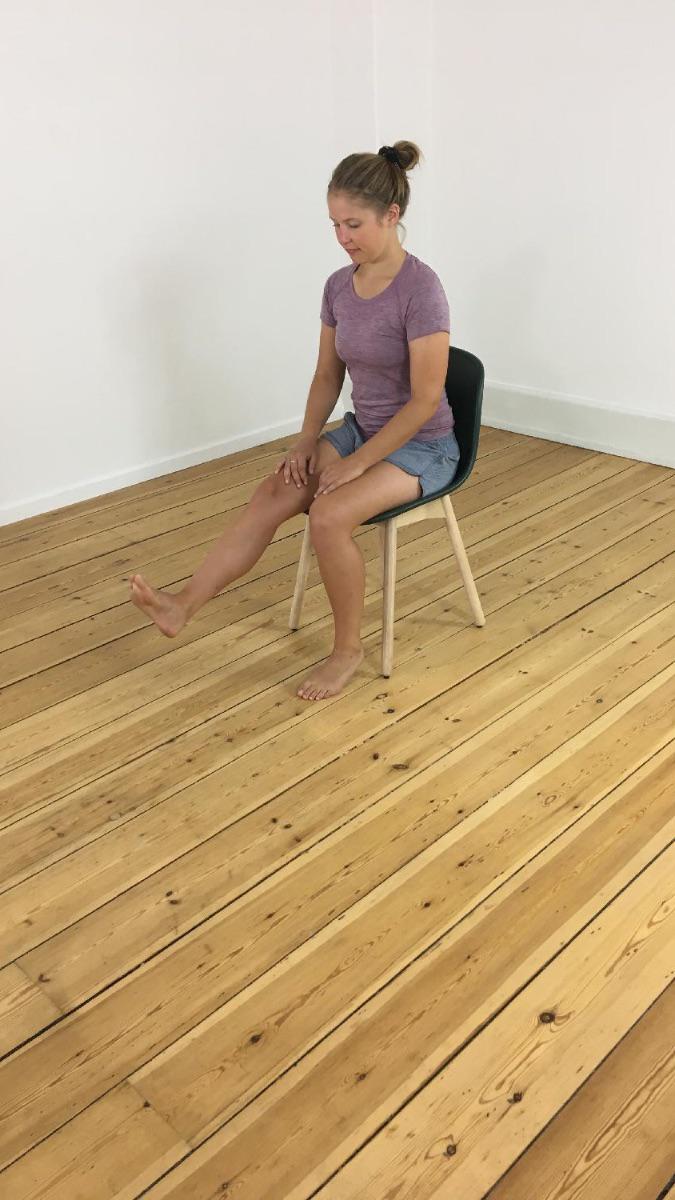 | **Stab. 1.2: Calf raises I**  **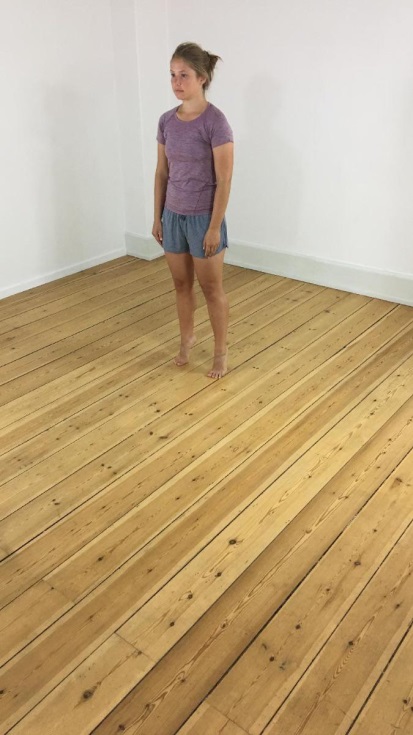** | **Strength 1.2 Lying ankle bends**  **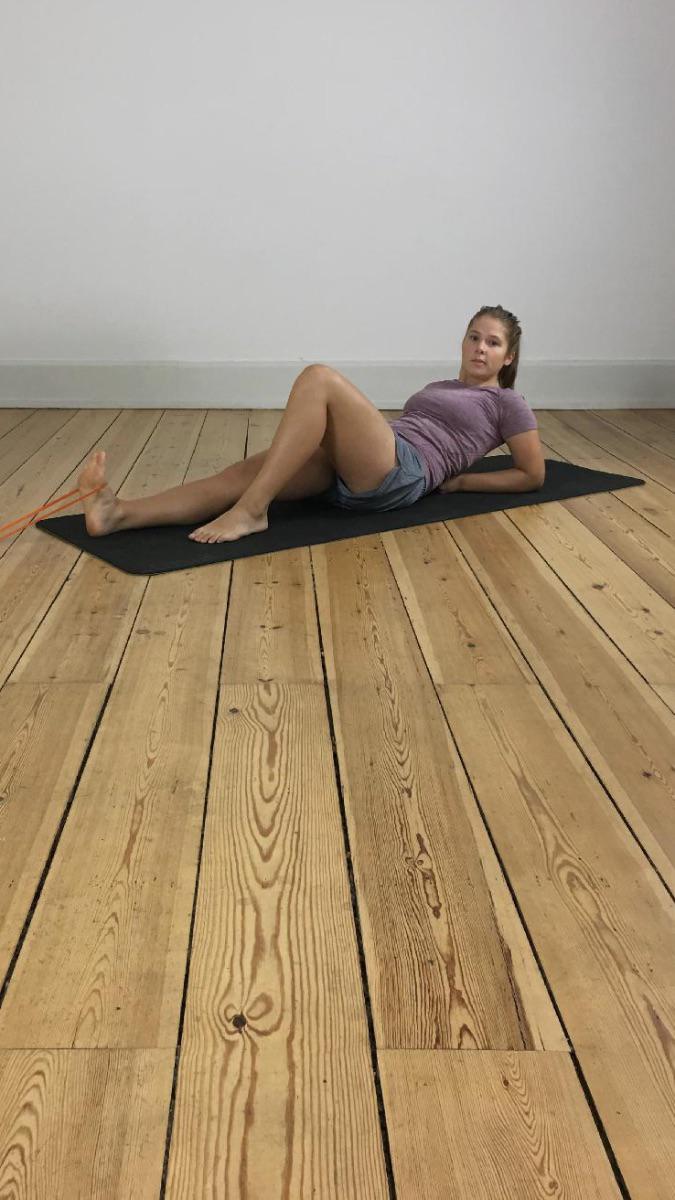** | **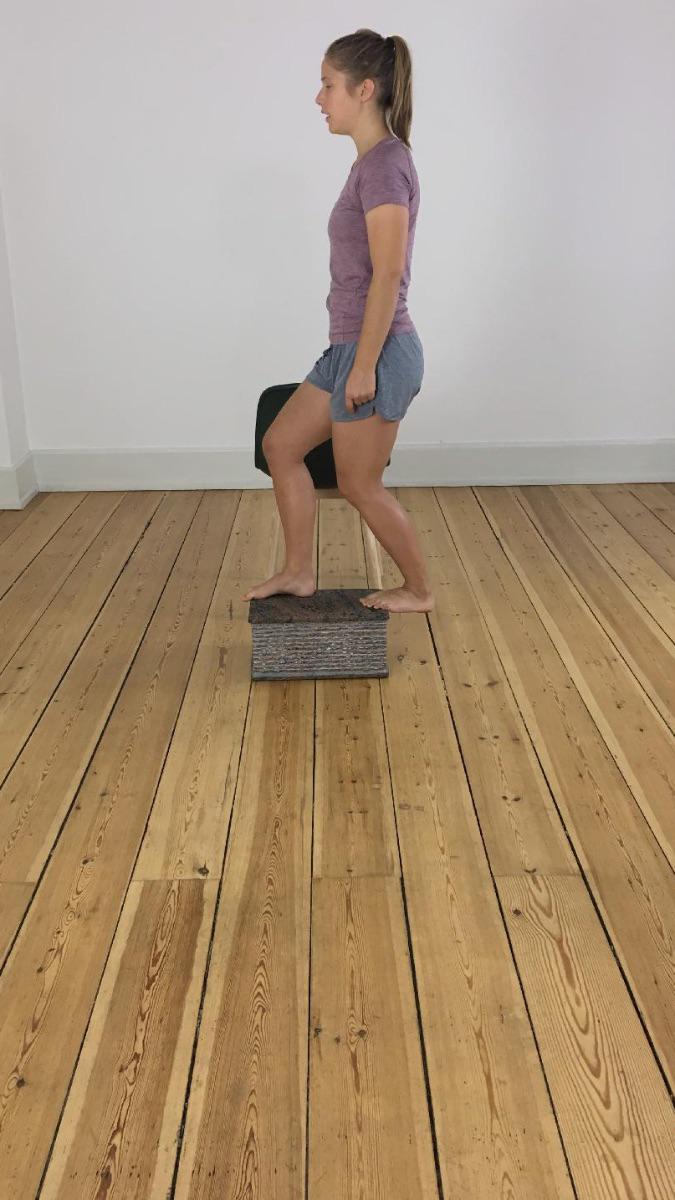Stretch 1.2 Bend knee Calf stretch.** |
| Sit on a chair. Lift the one foot from the floor. Tilt the foot outwards (eversion) and inwards (inversion) as far as possible  10 reps each foot. | **Level I:** Stand with shoulder width stance and the feet pointing forward. Raise the heels from the floor so that you’re on your tiptoes and return slowly.  10 reps.  **Level II:** Perform the exercise while looking from side to side. | Secure the rubber band on a radiator pipe or similar at ground level. Lie on your back with the injured leg extended. Place the end of the rubber band over the back of the foot just behind the toes. Bend the foot upwards while using the rubber band to manage resistance.  The rubber band needs to be so tight that it is only possible to perform 10 reps for 3 sets. | Stand on a stair step with one foot only touching the step with the forefoot and the heel free from the edge.  Lower the heel downwards with the knee bended until you feel a stretch in the calf muscles. Put as much weight on the leg as possible without provoking pain.  Keep stretch position 30 sec. x 3 reps. |
| 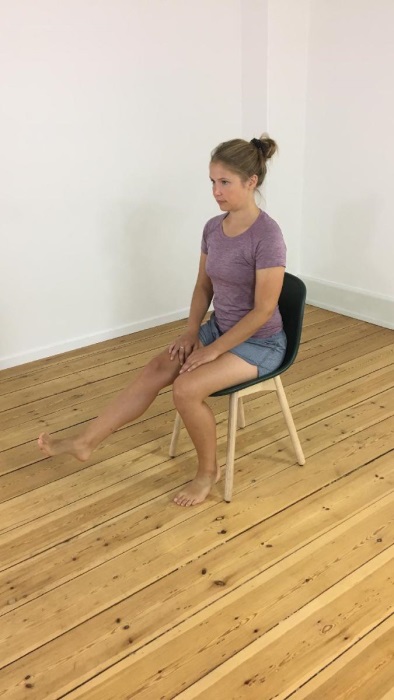Mob. 1.3: Ankle circles | **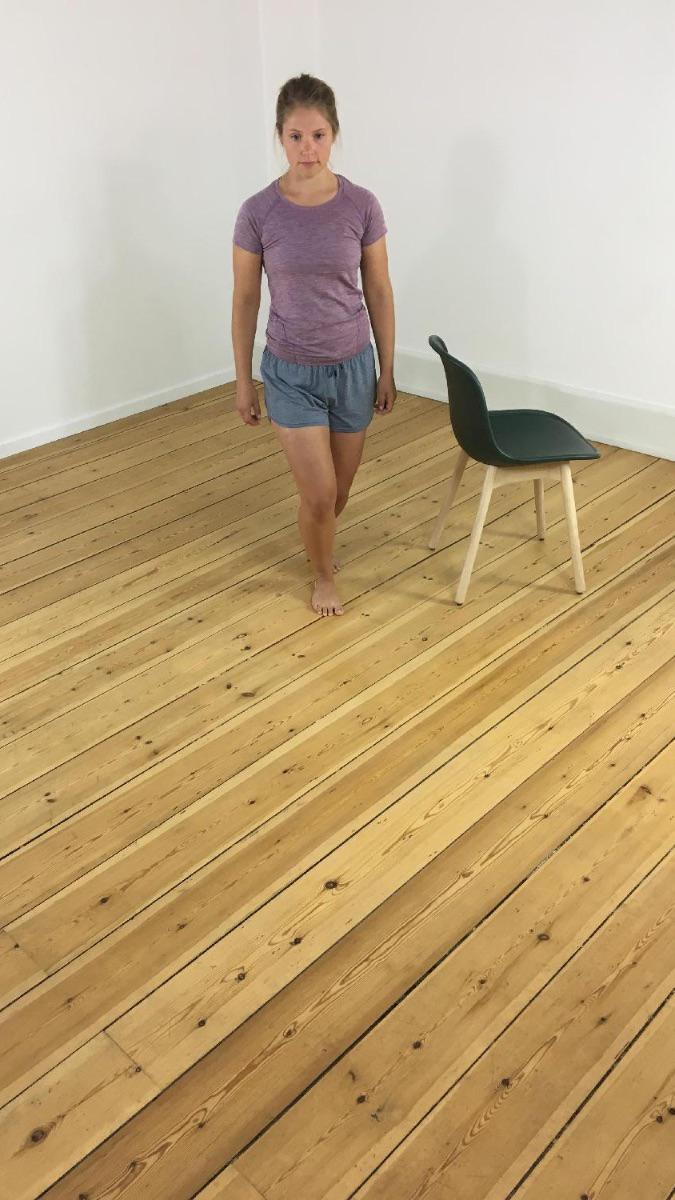Stab. 1.3: Split stance knee bends I** | **No further progression** | **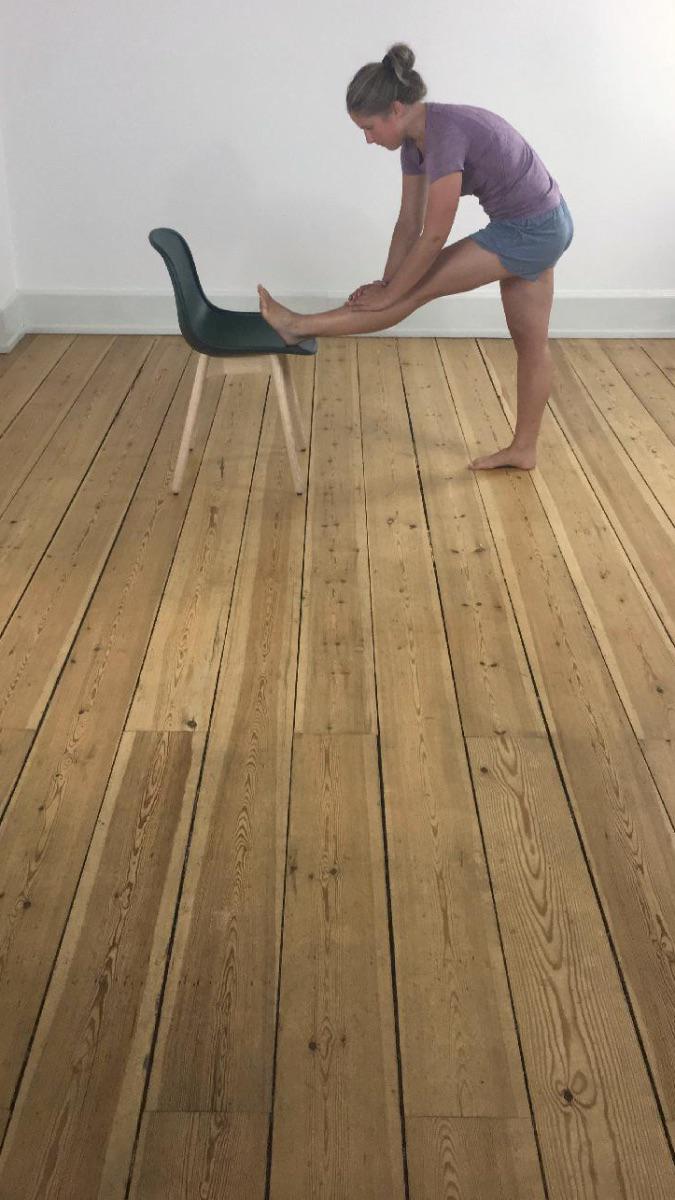Stretch 1.3: Hamstring stretch** |
| Sit on a chair. Lift the one foot from the floor. Turn the foot in large circles. Start with 5 rotations in a clockwise direction and then 5 rotations in the opposite direction.  Repeat twice | **Level I:** Stand with the feet in line with the injured foot in front.  Keep the balance while you slowly bend the knees beyond the toes as far as possible, so that the ankle bends to its maximum. You should feel it tightens in the back of the heel and clamps in the front, but it must not be painful.  10 repetitions.  **Level II:** Perform the exercise while looking from side to side. |  | While standing towards a chair put one heel upon the seat. Lower the upper body towards the elevated leg until it stretches in the hamstrings. Be careful not to hyperextend the knees.  Keep stretch position 30 sec. x 3 reps. |
| No further progression | 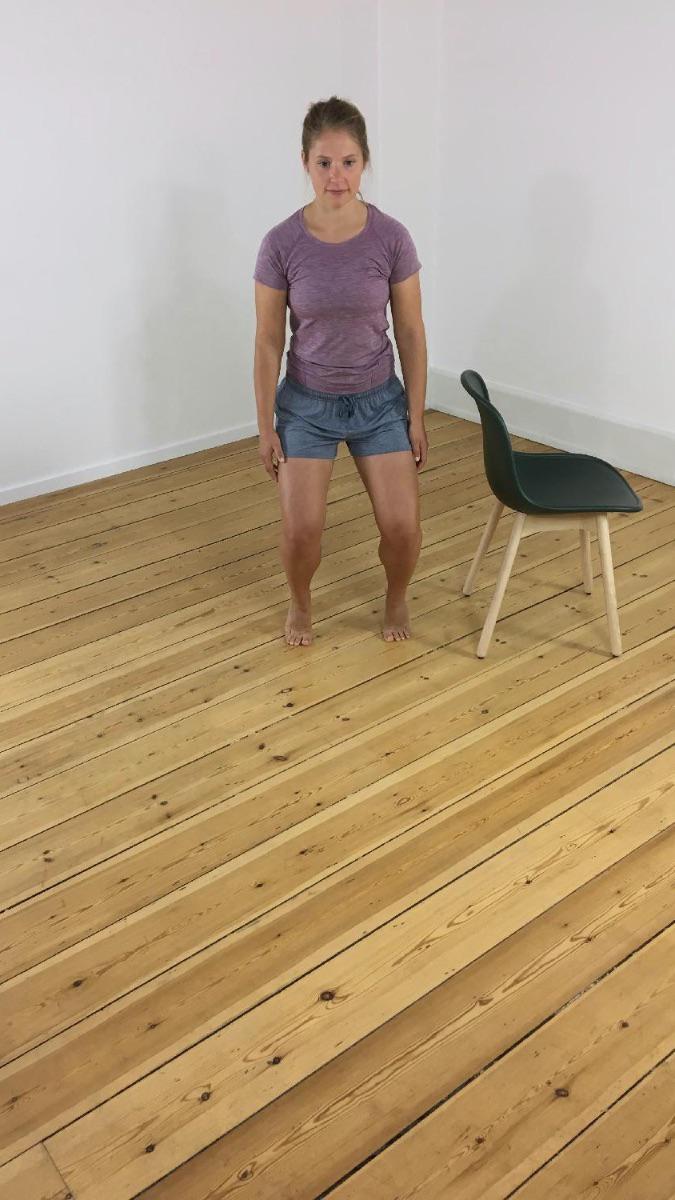**Stab. 1.4: Calf raise with knee bend I** |  | **No further progression** |
|  | **Leve I:** Stand with shoulder width stance and the feet pointing forward. Bend your knees as far beyond the toes as possible so that the ankles bend to their maximum. You should feel it tightens in the back of the heel and clamps in the front, but it must not be painful. While bending your knees raise your heels so that you’re on your tiptoes and return slowly.  Repeat 5 times  **Level II:** Perform the exercise while looking from side to side. |  |  |

Exercises in phase 2 of the program

| Phase 2 | | | |
| --- | --- | --- | --- |
| Mobility | **Stability/balance** | **Strength** | **Stretch** |
| 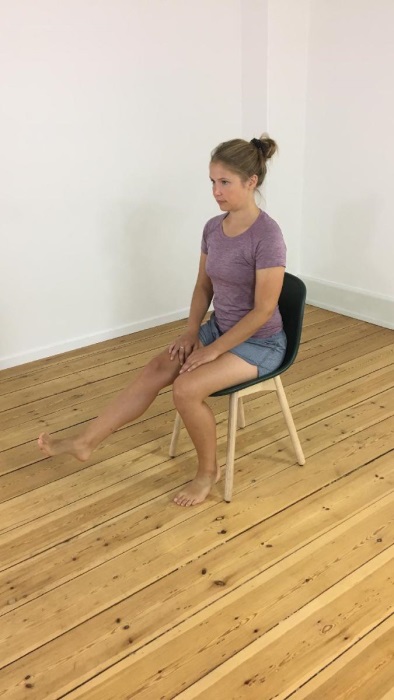Mob. 2.1: Ankle circles | **Stab 2.1: One leg balance I**  **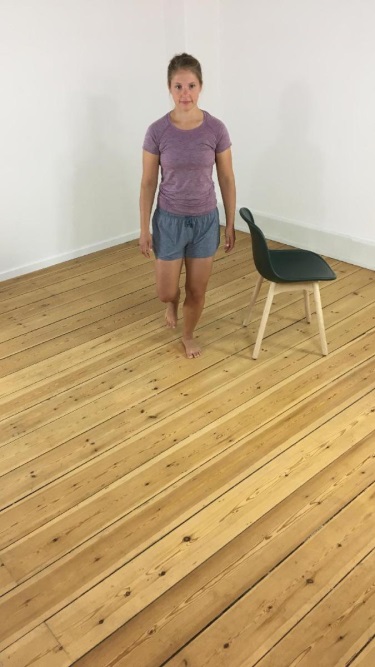** | **Strength 2.1: Sitting ankle inwards tilt**  **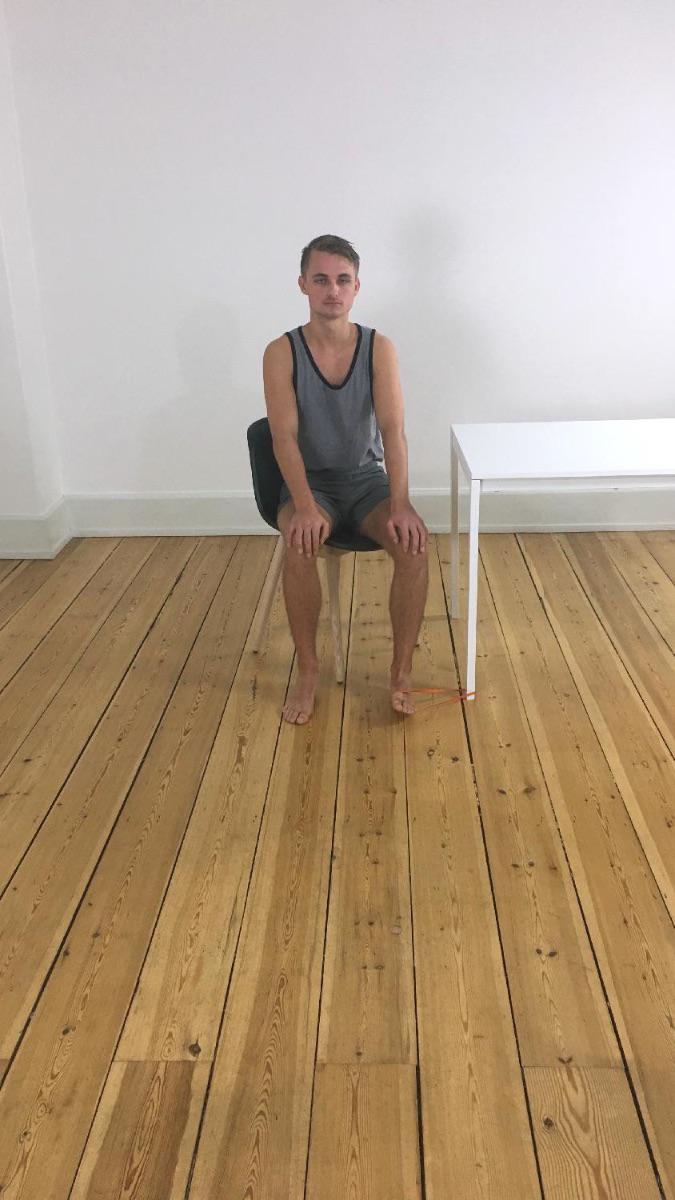** | **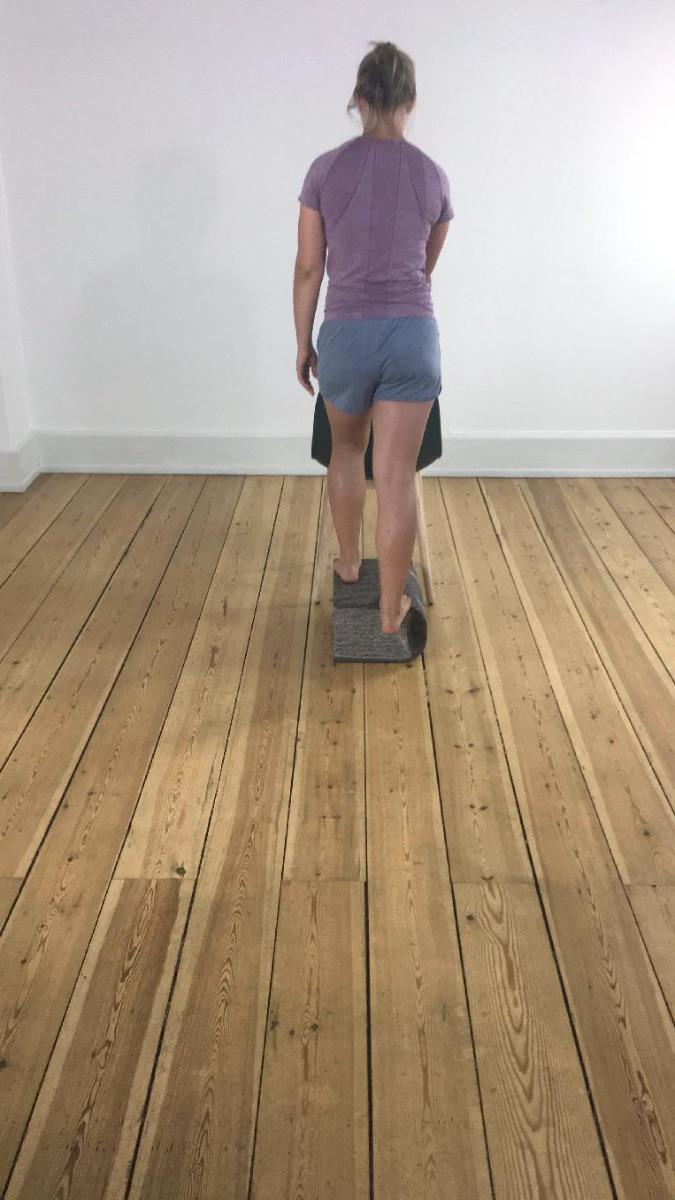Stretch 2.1: Straight leg calf stretch** |
| Sit on a chair. Lift the one foot from the floor. Turn the foot in large circles. Start with 5 rotations in a clockwise direction and then 5 rotations in the opposite direction.  Repeat twice | **Level I:** From a normal standing position on two legs, gradually place more weight on one leg and lift the other leg from the floor.  Bend the standing knee slightly and hold for 10 sec  3 reps. On each leg  **Level II:** Hold the balance for 20 sec.  **Level III:** Hold the balance for 10 seconds and look from side to side. | Sit on a chair with bended knees and a table leg beside you. Secure the rubber band on the table leg and place the other end on the forefoot just behind the toes. Slowly tilt your foot inwards and return. The heel should be kept on the floor through the movement. Tilt as much in both directions as possible. The knees and hip are kept fixed in the exercise.  The rubber band needs to be so tight that it is only possible to perform 15 reps for 3 sets. Complete the exercise with both legs | Stand on a stair step with one foot only touching the step with the forefoot and the heel free from the edge.  Lower the heel downwards with the knee extended until you feel a stretch in the calf muscles. Put as much weight on the leg as possible without provoking pain.  Keep stretch position 30 sec. x 3 reps. |
| No further progression | **Stab. 2.2: Calf raise on one leg**  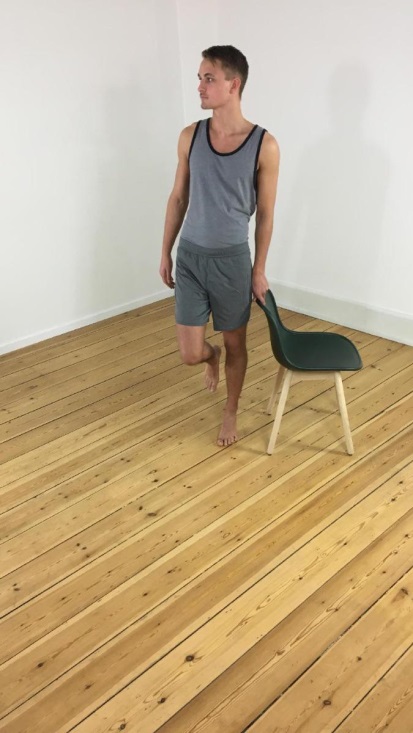 | **Strength 2.2: Sitting ankle outwards tilt**  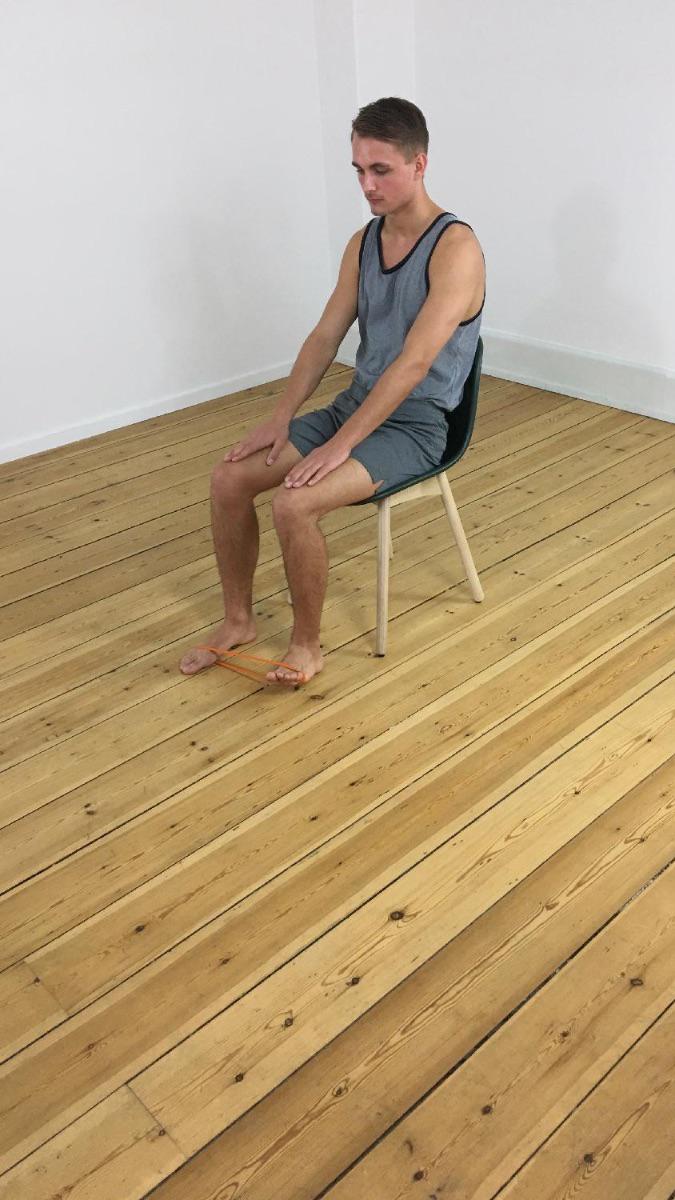 | 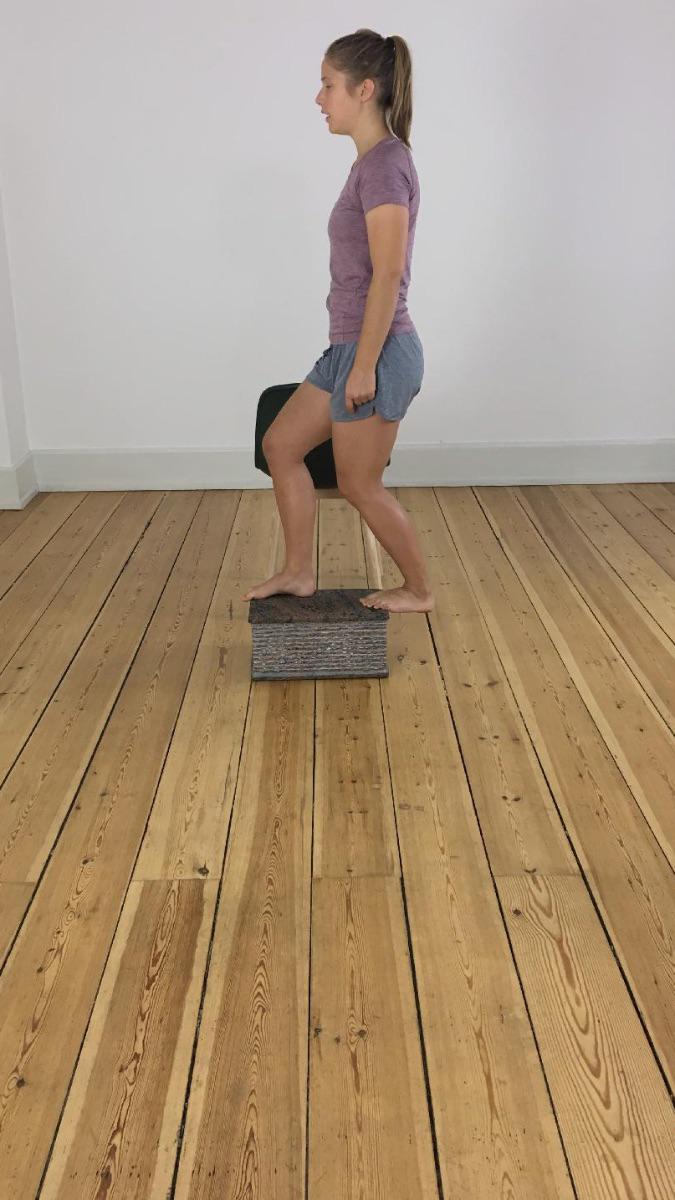**Stretch 2.2 Bend knee Calf stretch.** |
|  | **Level I:** From a normal standing position on two legs, gradually place more weight on one leg and lift the other leg.  While standing on one leg raise your heels so that you’re on your tiptoes. Hold the balance for a couple of seconds and return slowly.  Repeat 10 times  **Level II:** Perform the exercise while looking from side to side. | Sit on a chair with bended knees. Place the rubber band around both feet just behind the toes. Keep the unscathed foot steady while you slowly tilt the injured foot outwards and return. The heel should be kept on the floor through the movement. Tilt as much in both directions as possible. The knees and hip are kept fixed in the exercise.  The rubber band needs to be so tight that it is only possible to perform 15 reps for 3 sets. Complete the exercise with both legs | Stand on a stair step with one foot only touching the step with the forefoot and the heel free from the edge.  Lower the heel downwards with the knee bended until you feel a stretch in the calf muscles. Put as much weight on the leg as possible without provoking pain.  Keep stretch position 30 sec. x 3 reps. |
|  | 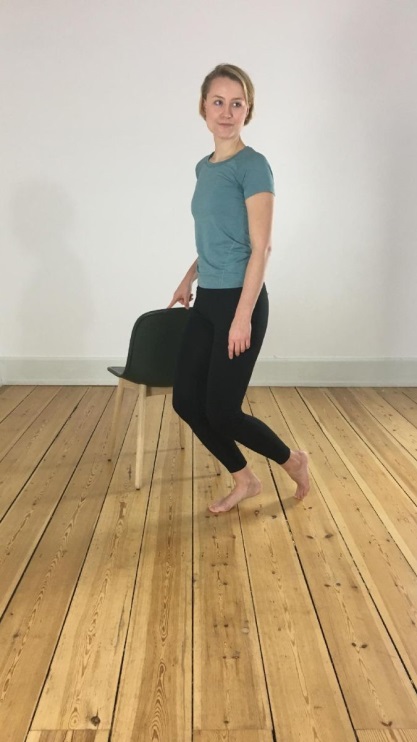**Stab. 2.3: Calf raise with knee bend.** | **No further progression** | 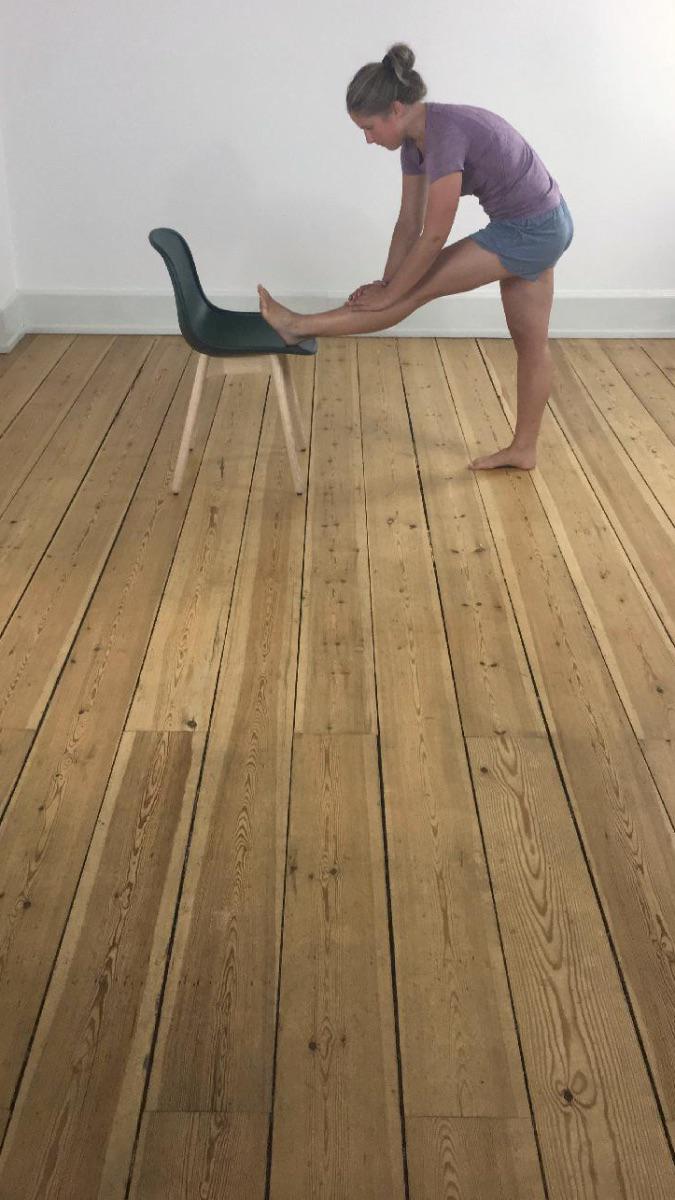**Stretch 2.3: Hamstring stretch** |
|  | **Level I**: From a normal standing position on two legs, gradually place more weight on the injured leg and lift the other leg. Move the unscathed foot behind the injured and slightly put some weight on the toes for balance. From this position bend the knee beyond the toes on the injured leg. While bending, raise your heel from the floor and slowly return. Complete the specified number of repetitions before switching to the other leg. To increase difficulty, look from side to side while performing the repetitions.  5 reps on each leg  **Level II**: 7 reps on each leg |  | While standing towards a chair put one heel upon the seat. Lower the upper body towards the elevated leg until it stretches in the hamstrings. Be careful not to hyperextend the knees.  Keep stretch position 30 sec. x 3 reps. |
|  | **No further progression** |  | **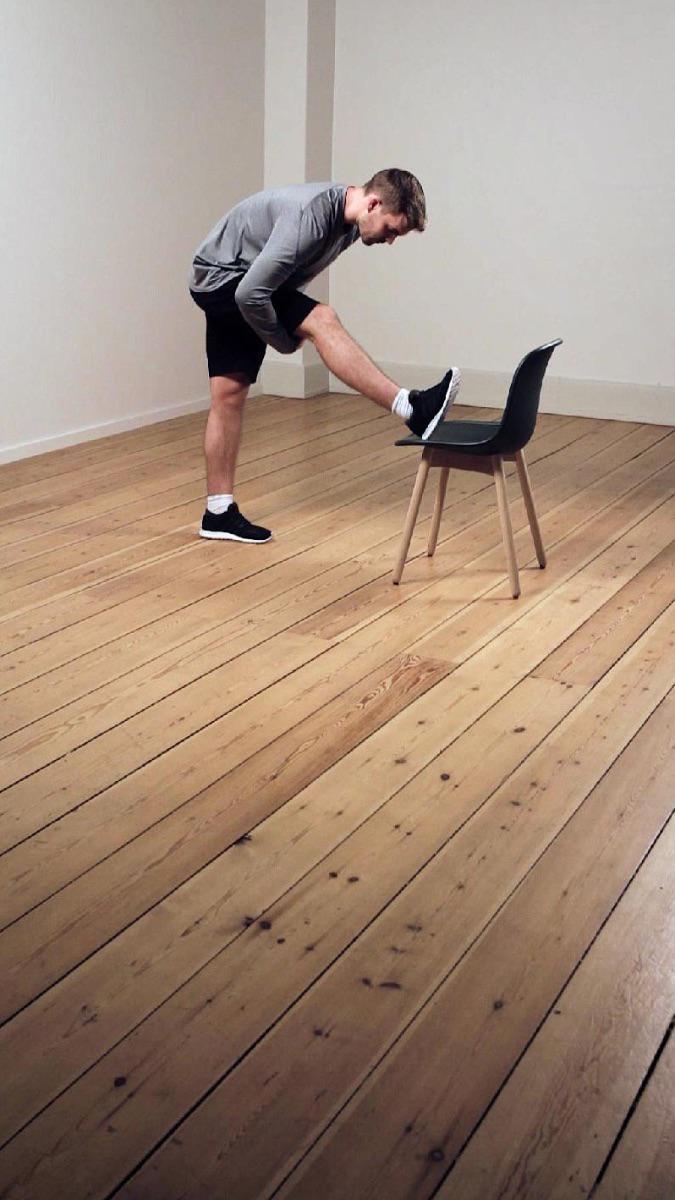Stretch 2.4: Bend knee hamstring stretch.** |
|  |  |  | While standing towards a chair put one heel upon the seat. Bend the elevated knee slightly while lowering the upper body towards the knee until it stretches in the hamstrings. You can place your arms under the thigh for better control of the bended knee.  Keep stretch position 30 sec. x 3 reps. |

Exercises in phase 2 of the program

| Phase 3 | | | |
| --- | --- | --- | --- |
| Mobility | **Stability/balance** | **Strength** | **Stretch** |
| 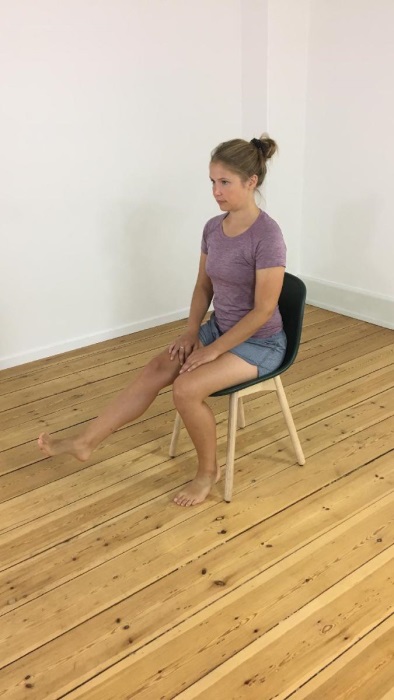Mob. 2.1: Ankle circles | **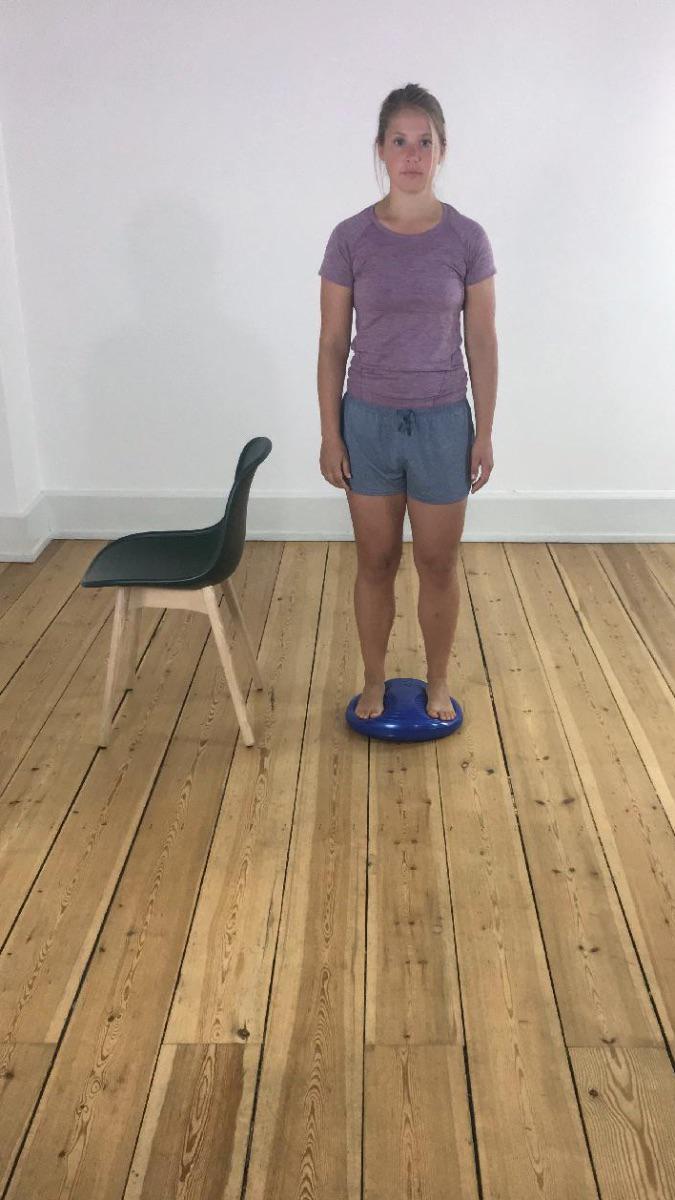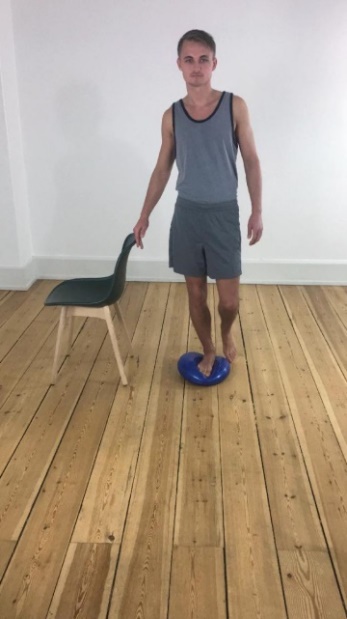Stab. 3.1: Balance on uneven surface**  **Level I Level II** | **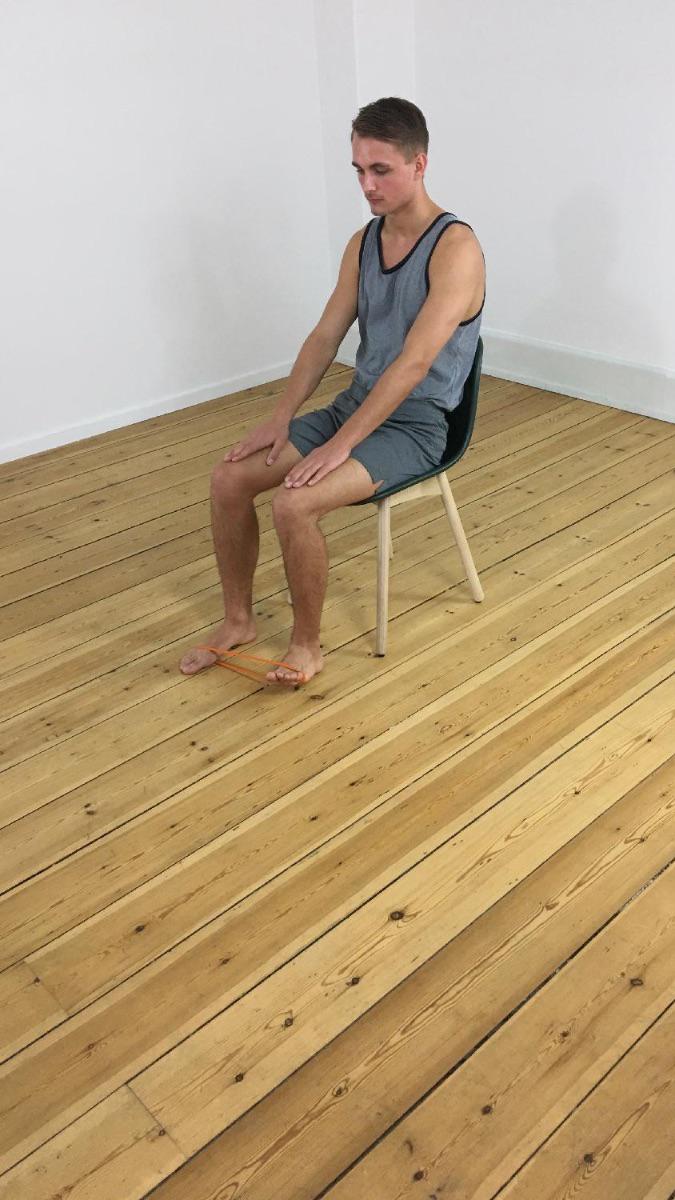Strength 3.1: Sitting ankle outwards tilt** | **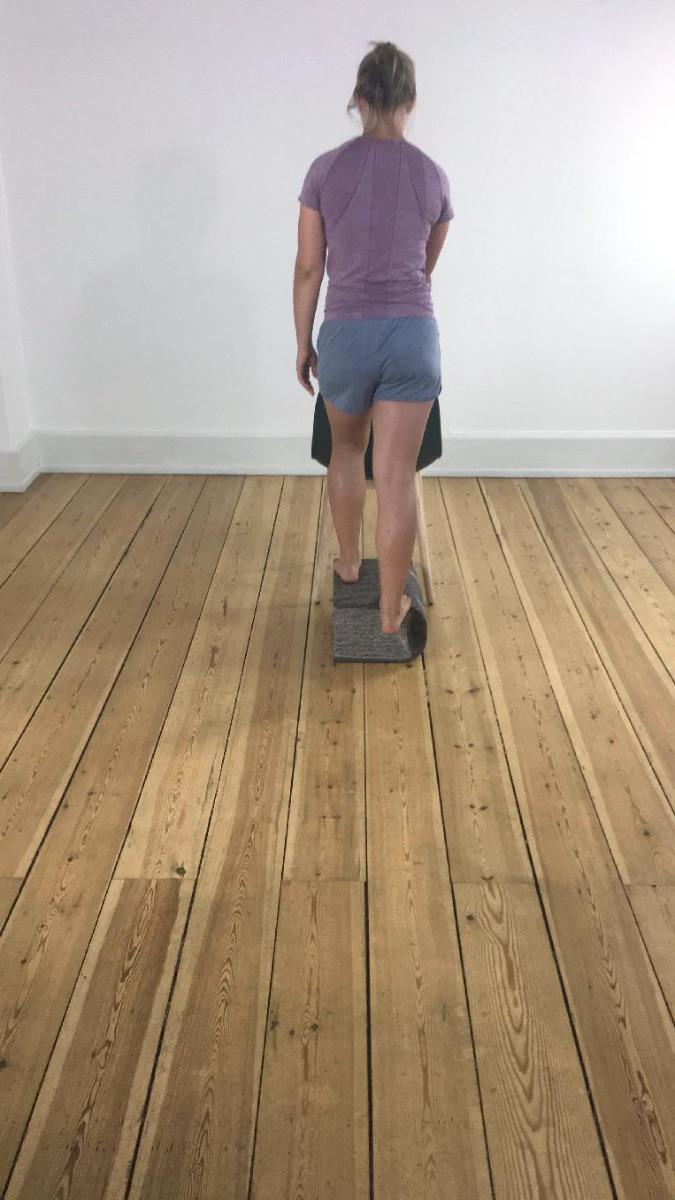Stretch 3.1: Straight leg calf stretch** |
| Sit on a chair. Lift the one foot from the floor. Turn the foot in large circles. Start with 5 rotations in a clockwise direction and then 5 rotations in the opposite direction.  Repeat twice | **Level I:** With a slightly bended knee stand on one leg on a balance board, hard pillow (like a couch pillow) or similar. Hold the balance without the other leg touches the ground. Repeat with the other leg.  Hold balance for 1 min. on each leg.  **Level II:** With slightly bended knees stand on both legs on a balance board, hard pillow (like a couch pillow) or similar. Slowly and with control shift your weight back on your heels and forth on your toes for 20 sec. Afterwards shift your weight from side to side for 20 sec.  Hold the balance in total for 1 min. | Sit on a chair with bended knees. Place the rubber band around both feet just behind the toes. Keep the unscathed foot steady while you slowly tilt the injured foot outwards and return. The heel should be kept on the floor through the movement. Tilt as much in both directions as possible. The knees and hip are kept fixed in the exercise.  The rubber band needs to be so tight that it is only possible to perform 15 reps for 3 sets. Complete the exercise with both legs | Stand on a stair step with one foot only touching the step with the forefoot and the heel free from the edge.  Lower the heel downwards with the knee extended until you feel a stretch in the calf muscles. Put as much weight on the leg as possible without provoking pain.  Keep stretch position 30 sec. x 3 reps. |
|  | **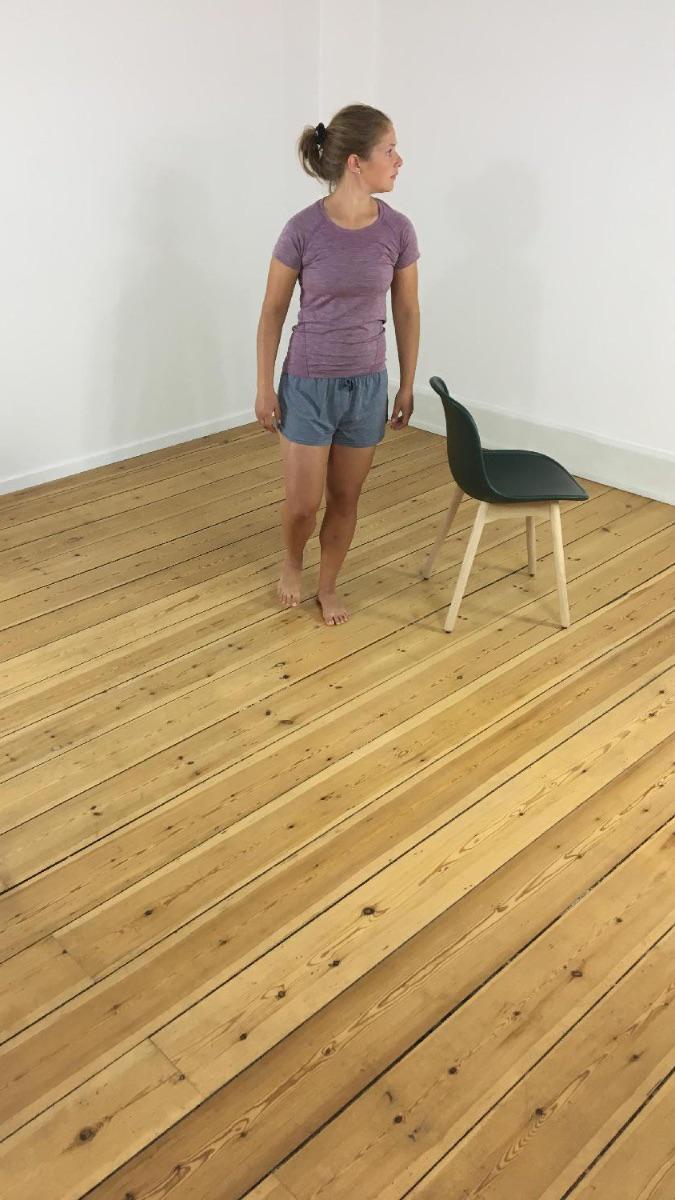Stab. 3.2: One leg balance II** | **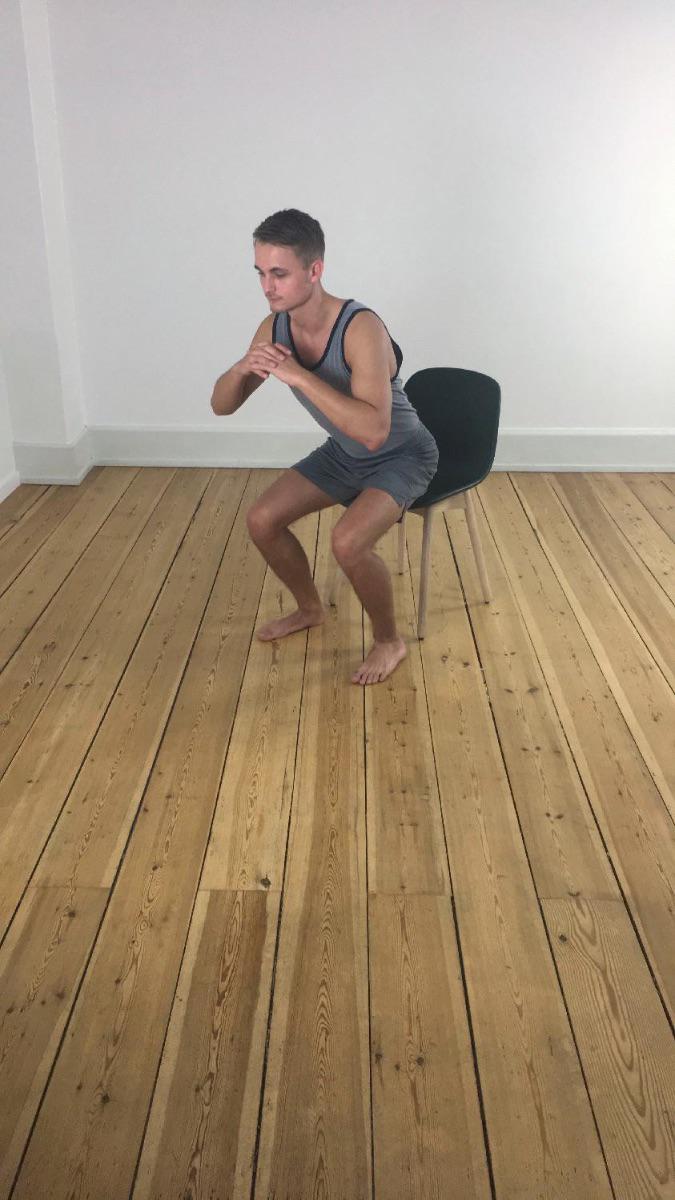Strength 3.2: Squat** | **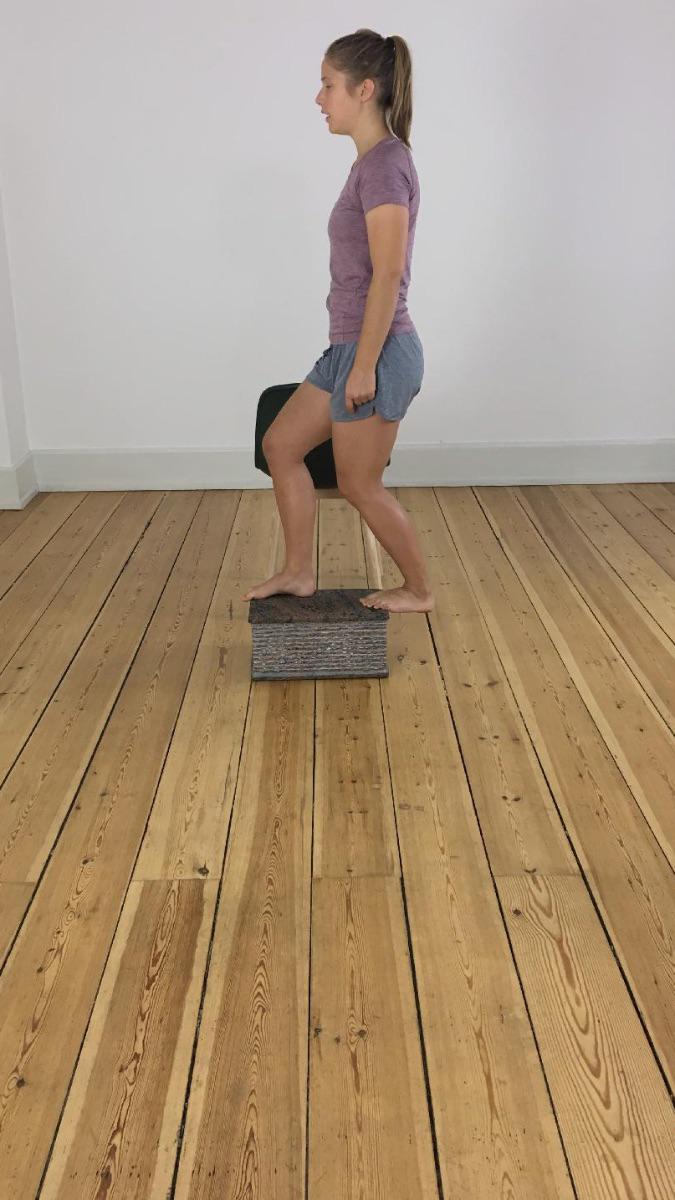Stretch 3.2: Bend knee Calf stretch** |
|  | From a normal standing position on two legs, gradually place more weight on one leg and lift the other leg from the floor.  Bend the standing knee slightly and hold for 10 sec while looking from side to side  3 reps. On each leg | Place a chair a hands length behind you and stand with shoulder width stance. Weight should be equally distributed on both legs.  Bend your knees as would you sit down on the chair but halt the movement just before you are sitting. Slowly return to upright position. Keep the knees oriented parallel with feet through the movement.  15 reps x 3 sets. | Stand on a stair step with one foot only touching the step with the forefoot and the heel free from the edge.  Lower the heel downwards with the knee bended until you feel a stretch in the calf muscles. Put as much weight on the leg as possible without provoking pain.  Keep stretch position 30 sec. x 3 reps. |
|  |  | **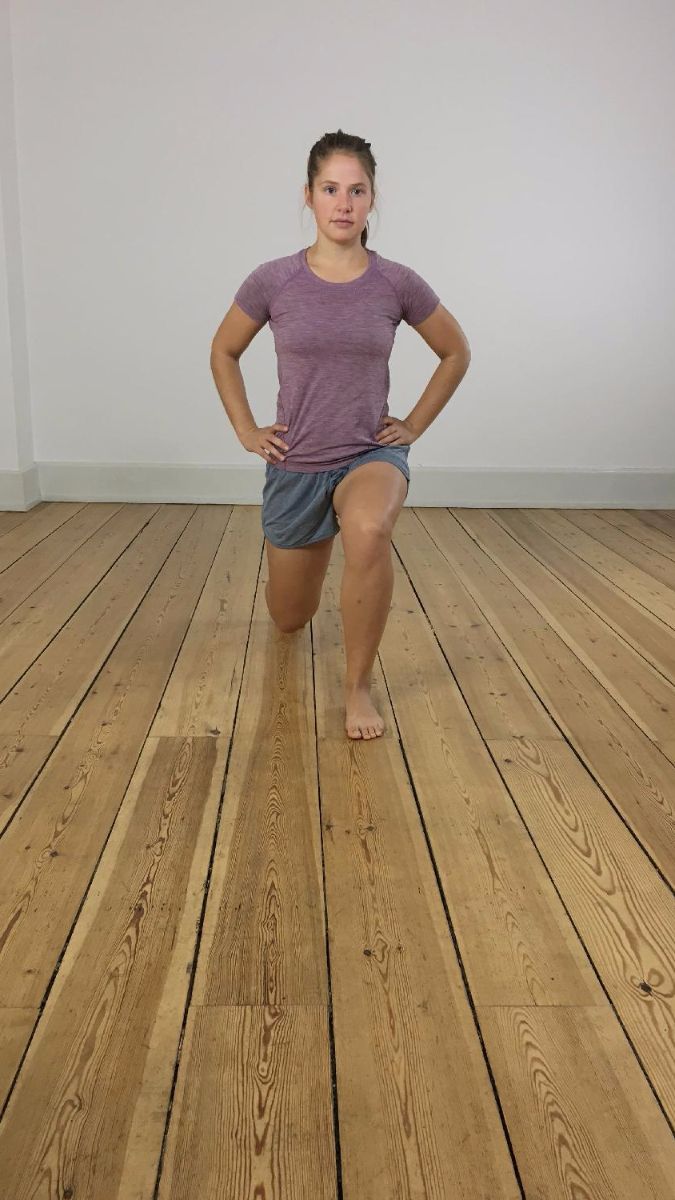Strength 3.3: Lounges** | **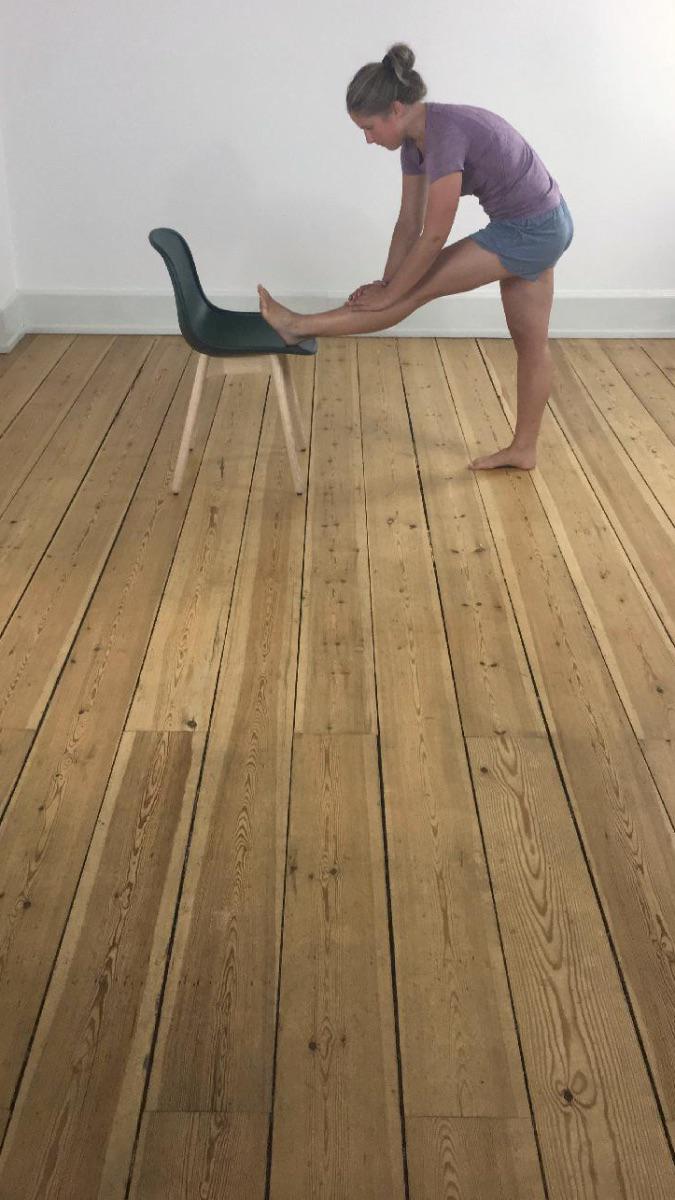Stretch 3.3 Hamstring stretch** |
|  |  | Stand with a shoulder width stance. Make a large step forward and lower your hips until both knees are bent at about a 90-degree angle and return to upright position. Make sure your front knee is directly above your ankle and keep your upper body straight with your shoulders relaxed.  Repeat 15 times and switch leg. 3 sets for each leg. | While standing towards a chair put one heel upon the seat. Lower the upper body towards the elevated leg until it stretches in the hamstrings. Be careful not to hyperextend the knees.  Keep stretch position 30 sec. x 3 reps |
|  |  | 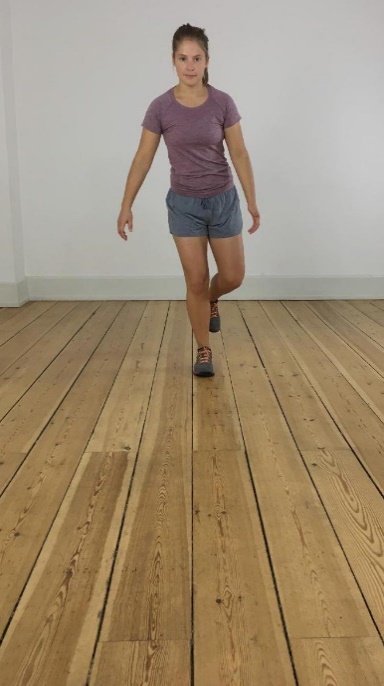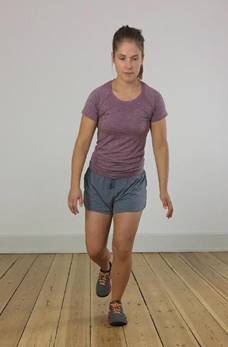**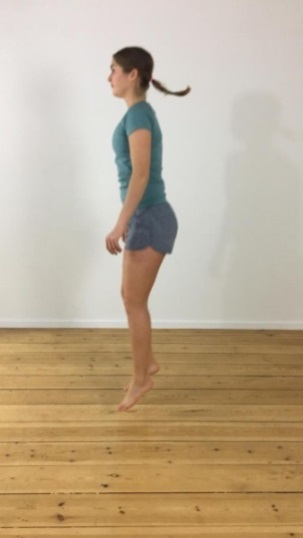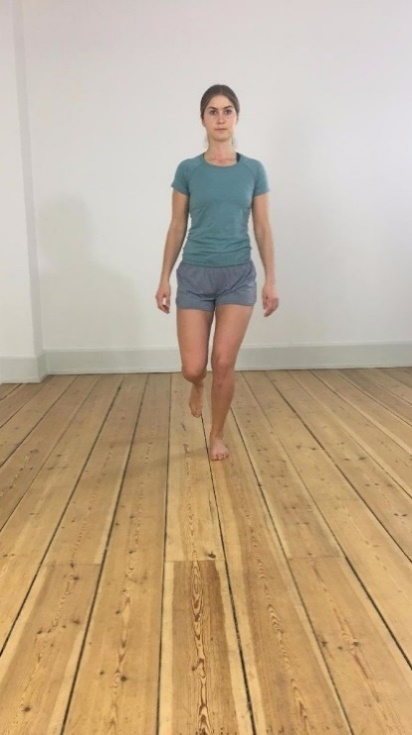Strength 3.4: Jumps**  **Level I Level II**  **Level III Level IV** | **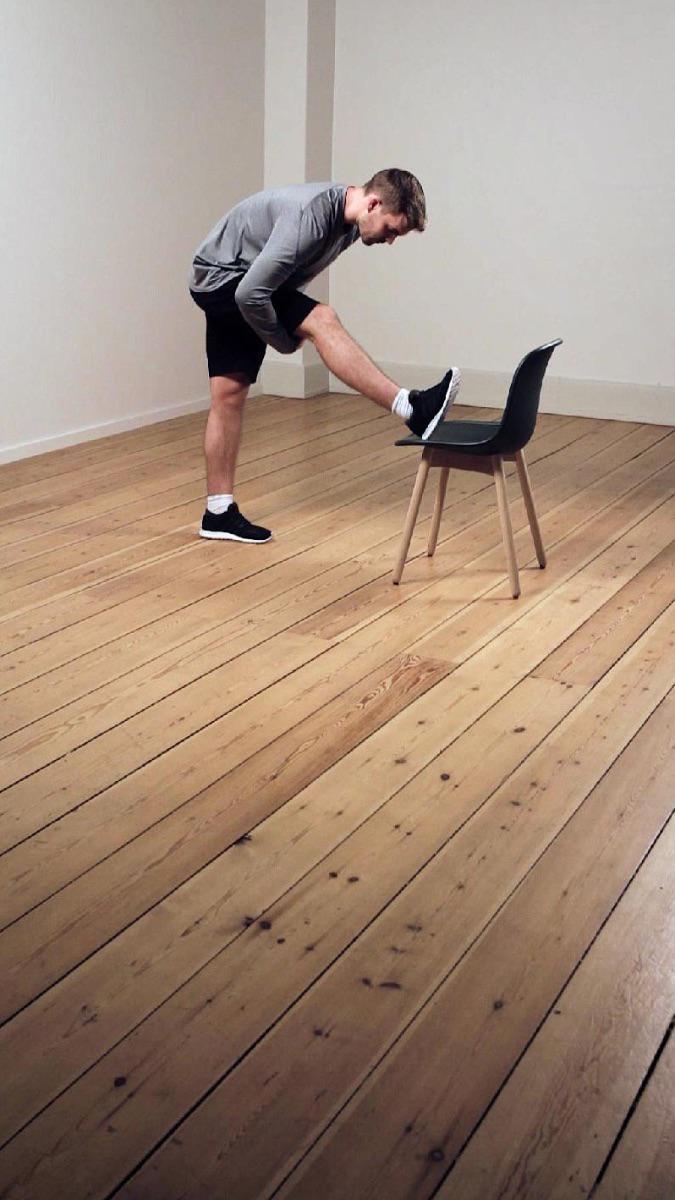Stretch 3.4 Bend knee hamstring stretch.** |
|  |  | **Level I:** **Straight jump**  Stand with a shoulder width stance. Jump straight upwards and land controlled with equal weight on both legs. Make sure that the knees are oriented over the foot in the landing, so that they do not fall inwards.  5 reps x 1 set  **Level II: One leg straight jump**  Stand on one leg. Jump straight upwards and land by controlling that the knee are oriented over the foot though the landing. The knee should not fall inwards. It should feel even on both legs.  5 reps on each leg.  **Level III: One leg forward jump**  Stand on one leg and jump back and forward. Find the balance on each landing before you jump again. It should feel even on both legs.  5 reps x 2 sets on each leg  **Level IV:** **One leg side jump**  Stand on one leg and jump from side to side. Find the balance on each landing before you jump again. It should feel even on both legs.  5 reps x 2 sets on each leg. | While standing towards a chair put one heel upon the seat. Bend the elevated knee slightly while lowering the upper body towards the knee until it stretches in the hamstrings. You can place your arms under the thigh for better knee control  Keep stretch position 30 sec. x 3 reps. |
