# Supplementary material for: Using the app “Injurymap” to provide exercise rehabilitation for people with acute lateral ankle sprains seen at the Hospital Emergency Department–A mixed-method pilot study
Source: PLOS Digit Health. 2023 May 15;2(5):e0000221. doi: 10.1371/journal.pdig.0000221 (PMC10184914; doi:10.1371/journal.pdig.0000221)
Supplement: S1 Fig — (DOCX) [file pdig.0000221.s003.docx]

#

S1 Fig: Number of completed exercise sessions per participant. EXses = exercise sessions.
