# Supplementary material for: Using the app “Injurymap” to provide exercise rehabilitation for people with acute lateral ankle sprains seen at the Hospital Emergency Department–A mixed-method pilot study
Source: PLOS Digit Health. 2023 May 15;2(5):e0000221. doi: 10.1371/journal.pdig.0000221 (PMC10184914; doi:10.1371/journal.pdig.0000221)
Supplement: S2 Fig — (DOCX) [file pdig.0000221.s004.docx]

## S2 Fig: Adherence by different grouping variables. This exploratory analysis of the adherence by group was conducted after the planned analysis and contains small and different sized groups. Interpretations should be done with caution. Participants with a bachelor degree or higher completed on average 15 exercise sessions, and participants with a shorter education completed on average 8 exercise session. Participants 50 years of age or more completed on average 22 sessions and participants with ages from 10 to 18 yr completed on average 4 sessions.

| Baseline charateristic | Item | N participants | Completed exercise sessions (mean) |
| --- | --- | --- | --- |
| Education level | Shorter than bachelor | 27 |  |
|  | Bachelor or higher | 29 |  |
| Age group | 10-18 | 9 |  |
|  | 19-34 | 24 |  |
|  | 35-49 | 19 |  |
|  | 50-65 | 8 |  |
| Sports active | Sports active | 12 |  |
|  | Not sports active | 10 |  |
| Work demands | Mostly sitting | 12 |  |
|  | Equal sitting and walking | 13 |  |
|  | Mostly walking | 10 |  |
