# Supplementary material for: Using the app “Injurymap” to provide exercise rehabilitation for people with acute lateral ankle sprains seen at the Hospital Emergency Department–A mixed-method pilot study
Source: PLOS Digit Health. 2023 May 15;2(5):e0000221. doi: 10.1371/journal.pdig.0000221 (PMC10184914; doi:10.1371/journal.pdig.0000221)
Supplement: S1 Text — (DOCX) [file pdig.0000221.s005.docx]

# S1 Text: Description of the exercise program.

**Reading guideline:** This exercise program is reported according to the Consensus on Exercise Reporting Template (CERT) [1].

**Materials:** circular rubber band, chair, table, stair step, balance board or pillow.

**Provider:** The exercises are available in the app “InjuryMap”. The program has been developed by two rheumatologists and reviewed by two physiotherapists. All experts had experience in treating ALAS patients. The exercise program was then compared and adjusted to current evidence in exercise rehabilitation for acute ankle sprains.

**Delivery:** It is possible to perform the exercises unsupervised at home. However, participants are free to perform the exercises anywhere they see fit. Participants are encouraged to adhere to the program by reminder notifications on their phone and by the exercise progression. The progression was designed so that participants need to “complete” a number of sessions to be able to progress to more challenging exercise. This resembles some game types and might motivate participant in completing exercise sessions. Data of exercise completion are saved in the app and used to analyze adherence. With each exercise the participants must answer pain level and difficulty. If the participants record no or low pain and low difficulty in an exercise, the app chooses a progression of the exercise next time the participants begin a training session.

**Description:** The exercise program consists of three phases with increasing difficulty. **Phase 1** focuses on stimulating ankle mobility without provoking the acute injury. The exercises are low load with respect for end range motions. **Phase 2** focuses on increasing balance and ankle stability. The phase includes several weight bearing exercises but no impacts. **Phase 3** focuses on strength and mobility. The goal is to return participants to normal activity level. The phase includes strength exercises and jumping exercises with change of direction.

Each phase consists of several exercise categories. The categories are 1) mobility, 2) stability/balance, 3) strength and 4) stretching. Within each phase there can be more than one exercise in each category. An exercise session will consist of minimum one exercise from each category, but not necessarily all exercises in a category. Exercises can also contain a number of difficulty levels. All exercises in each category must be completed on their highest difficulty before a participant can progress to the next phase. The exercises can potentially cause some discomforts or pain and participants are advised that increased swelling after training session or increased pain until next day should not be tolerated.

A detailed description of the specific exercises in each phase can be found in S2 Table 1.

[1] Slade SC, Dionne CE, Underwood M, Buchbinder R: Consensus on Exercise Reporting Template (CERT): Explanation and Elaboration Statement. *Br J Sports Med* 2016.
