# Supplementary material for: Using the app “Injurymap” to provide exercise rehabilitation for people with acute lateral ankle sprains seen at the Hospital Emergency Department–A mixed-method pilot study
Source: PLOS Digit Health. 2023 May 15;2(5):e0000221. doi: 10.1371/journal.pdig.0000221 (PMC10184914; doi:10.1371/journal.pdig.0000221)
Supplement: S3 Text — (PDF) [file pdig.0000221.s007.pdf]

Study protocol

Title: A pilot study investigating the feasibility and preliminary efficacy of the app “Injury-map” in providing exercise rehabilitation for persons with acute lateral ankle sprains seen at the Emergency Department – a mixed-method pilot study.

Trial registration: NCT03550274.

Background.

Acute ankle sprains account for 4-5% of all Emergency Department visits in Denmark, equating to approximately 120 visits per day [1]. The number of injuries in the emergency department is, however, a potential strong underestimating of the actual number of injuries since under 50% of persons suffering from acute ankle sprains seeks formal care [2, 3]. Approximately 91-96% of ankle related sprains are acute lateral ankle sprains (ALAS) [4, 5]. ALAS has been recognized as one of the most frequent types of injuries in several sports disciplines [2, 6]. ALAS is highest in indoor/court sports with incidence rates estimated as 7 per 1000 exposures [7] and with re-injury occurrence of 34% [8]. ALAS is often regarded as an innocuous injury [4] and especially health professionals tend to overestimate the recovery [9]. However, 32-74% of individuals with a history of lateral ankle sprains have prolonged symptoms such as pain, decreased function and subjective instability for several years after their initial injury[2, 8]. These factors highlight the importance of a focus on ALAS from both health professionals and persons with ankle sprains.

The financial impact of ALAS is high [5] but exercise therapy has proven to be a cost-effective rehabilitation in treating ALAS [10-13] and in preventing re-injury [11, 14, 15]. Regardless, few patients are prescribed exercise programs or physiotherapy and the vast majority of expenses (>88%) concerning ALAS are spent on diagnosis [4]. The current standard care for ALAS in the Emergency Department is either no exercise instructions or a simple pamphlet of home-based exercises. Systematic exercise programs are rarely prescribed and patients are often left to their own ability to find information.

Modern technology has the potential to be a powerful tool in providing easily accessible exercise programs and in solving the current enigmas of exercise research regarding exercise habits [16]. Applications for smart devices (apps) have the ability through interaction between users and smartphone to tailor specific information and real-time monitoring in exercise rehabilitation [16]. A serious challenge in the use of apps is that there is a general lack of evidence-based solutions and healthcare apps often wrongly claim to be evidence founded [17-19]. Because of the availability of health-apps, this poses a major problem that the community needs to address [18].

Injury-map© is an exercise app designed for treating different musculoskeletal problems including ALAS. The exercise program has been developed by health professionals and has the potential to provide an easy-accessible management of ALAS rehabilitation. However, the app has currently not been tested in a clinical trial with patients suffering from ALAS. . Before undertaking such a large scale trial work, we wish to pilot test the app.

Aim

The aim of this pilot study is to investigate the feasibility and preliminary efficacy of an exercise program on a mobile device in patients with ALAS when seen in the Emergency Room at a public hospital. This pilot work is undertaken to inform a subsequent large-scale clinical trial given that feasibility is indicated.

Trial design

This is a mixed method pilot cohort study. The SPIRIT checklist [20] for trial protocols and the PREPARE trial guide [21] has been used for the overall framework of this paper.

Methods

Study setting and recruitment

Participants will be recruited from the Emergency Department (ED) at Hvidovre Hospital by a qualified health specialist. The hospital is a public hospital and patients are covered by the Danish healthcare system. At the hospital, the current practice for non-surgical management of ALAS patients is RICE (Rest, Ice, Compression, Elevation) recommendations, mobility exercises and slowly return to activity. The current practice does not involve a systematic evidence-based exercise program with focus on progression, duration and dose. The exercise intervention in this study will be an evidence-based ad-on to normal care.

Recruitment will be done on different days (workdays, weekend) and different time intervals at the day, for the purpose of getting a heterogeneous ALAS group.

The exercise program will be available on any mobile device and/or tablet using Android or iOS. It is expected that patients primarily will exercise at home. However, they will not be restricted from seeking additional supervised care and exercises can be performed parallel with other treatment plans

Table 1: Eligibility criteria

Inclusion	Exclusion
- Acute lateral ankle sprain (< 48 hours) evaluated by a relevant health specialist in a hospital emergency room	- Fracture of the leg or foot (Ottawa rules and/or x-ray)<br>- Previous surgery in the foot/ankle or as a consequence of the current ankle distortion.<br>- Other serious injuries to the body in relation to ankle injury incidence.<br>- Serious illness.<br>- Not owning a smartphone or tablet.<br>- Unable to understand Danish.

Intervention

InjuryMap© offers a variety of feedback-adjustable exercise programs specifically designed for different regions and musculoskeletal conditions. The app requires user-registrations and a monthly paid subscription fee to access the exercise program. The participants in this study will not be charged for using the app.

The exercise program consists of three phases with increasing difficulty. Each phase consists of several categories of exercise types. The categories are 1) mobility, 2) stability/balance, 3) strength and 4)

stretching. Within each phase, there can be more than one exercise in each category. This makes it possible to adjust the difficulty of the exercises depending on user-feedback.

A comprehensive description of the exercises can be found in Appendix A

The exercise program is set up so several exercises must be completed subsequent each other in order to successfully complete a training session. Each exercise is accompanied by an explanatory video and the number of repetitions to be performed is written on the display. With each exercise, the participants must answer pain level and difficulty. If the participants record no or low pain and low difficulty in an exercise, the app chooses a progression of the exercise next time the participants begin a training session. The app encourages participants to complete exercise sessions three times a week, however, there are no formal limitation in how many times a week an exercise session can be done. Furthermore, are there not defined a number of weeks within the program must be completed.

In the exercise program, a circular rubber band is used in several exercises. The rubber band is a common and cheap product available in most sports stores. In this study, the rubber band will be delivered to the participant at the emergency room by the recruiting health specialist.

The app can be set up with a daily reminder notification to perform exercises and every week to register pain level. The spoken and written language in the application is Danish.

Outcome measures

The main outcomes related to feasibility:

If available, a pre-described evidence-based threshold for adequate feasibility will be defined for selected outcomes. The main outcomes for feasibility are:

- Retention rates: number of potentially eligible participants at the ED, number of persons contacted by the health specialist for information about the study, number of participants willing to participate, number of persons completing follow-up assessment. A loss to follow-up under 20% for persons willing to participate is considered adequate.
- Compliance to the exercise intervention. A compliance rate of >75% for the participants commencing the exercise intervention is considered adequate. Full compliance to the exercise will defined as; completing a minimum of 32 sessions, for a minimum of 3 times per week for 8 weeks. Furthermore, compliance to a minimum intervention will be defined as; completing a minimum of 12 sessions, for a minimum of 3 times per week for 4 weeks.
- Satisfaction with intervention

The main outcomes related to preliminary efficacy of the intervention are:

- Pain 0-100 modified VAS scale (mm.)
- Return to Activity (number of days)
- Re-injuries (number)
- Feeling of stable/unstable ankle 0-10 (points).

All outcomes will be collected in a written or oral format by SMS, email, app or phone. No physical test will be performed. A complete list of the outcomes in this study can be found in Appendix B.

Recruitment

One or more health specialist associated with the ED and responsible for ankle examinations will be identified for the purpose of recruiting participants to the study. Baseline characteristics will be collected at the ED by the health specialist with a written questionnaire together with an access code and download explanation for the exercise app. All eligible patients willing to participate will receive the app-based exercise intervention. Eligible persons who are not willing to participate will be given an opportunity to fulfill the baseline questionnaire.

Intervention outcomes will be collected continuously through the intervention period both by the exercise app and by a weekly string of SMS. A follow-up assessment of feasibility will be sent by e-mail eight weeks after the ED visit. Furthermore, after eight weeks a strategical selection of participants will be identified for a semi-structured interview. The interview will be done by phone.

As the app is built on an evidence-based exercise program, we expect no adverse events. Nonetheless, all potential adverse events will be registered. Participants will receive a weekly SMS where adverse events will be reported as discomforts or injuries obtained in relation to conducting the exercise program.

Statistical analysis

Baseline characteristics will be summarized with suitable descriptive statistics. Tests for normal distribution will be a consensus of the Shapiro-Wilk test and the quantile-quantile plot.

For the aim of investigating the feasibility, the loss to follow-up and the compliance will be compared to the previously described thresholds. Other feasibility outcomes will be summarized and discussed against comparative literature. Any adverse events will be addressed individually.

Intervention outcomes will be summarized and tendencies in treatment efficacy will be compared with comparable literature, however no formal hypothesis testing will be performed.

Interviews will be recorded and transcribed verbatim. There will be used a thematic approach. Differences between participants who complete the intervention and those who do not will be illustrated with phrases and discussed.

Participant timeline

It is expected that enrollment will be done from February 2018 to May 2018. The last follow-up is expected done ultimo June 2018. These time intervals might be adjusted slightly based on process development. The intervention period will be 8 weeks from ED discharge. Follow-up assessment will be conducted in the 9th week from discharge.

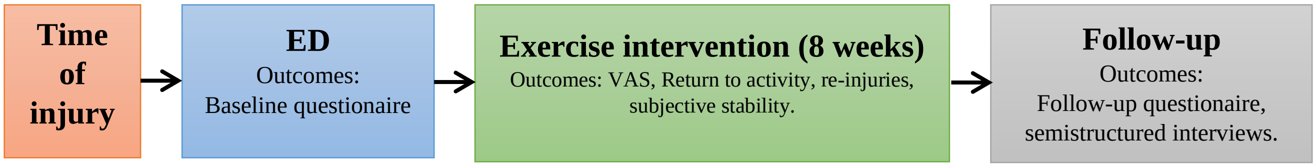

Figure 1: Participant flowchart. An elaborated Participant flowchart can be found in Appendix C

Sample size

The aim of this pilot study is to investigate the feasibility and preliminary efficacy and therefore no formal sample size estimation will be performed. A pragmatic number of approximately 60 people will be recruited for this project. We expect that 30 people will download the app and commence the exercise program

Controlling for bias: blinding etc.

Personnel with no other involvement in the study will perform recruitment. The research assistant responsible for the project execution will perform the outcome assessment, follow-up assessment and data analysis.

Ethics and dissemination

The Danish National Committee on Health Research Ethics will be contacted for approval. Informed written consent from the participants will be registered in connection with the recruitment at the ED. A participant can withdraw from the study at any time, without any consequences. All personal data will be handled according to the Danish act concerning processing of personal data. InjuryMap has previously been approved for handling personal data (file no. 2016-42-3535, Danish Data protection Agency). The study will follow the principles of the Helsinki declaration. The research department of Hvidovre hospital and the research assistant has no affiliation with InjuryMap© and no economic interest in the exercise app. A protocol will be registered at clinicaltrials.gov and all described outcomes will be published.

References

1. The Danish National patient registry L: **DS93 - Luksation og distorsion af led og ligamenter i ankel og fod**. In: *2015 - 2016.* www.esundhed.dk; 2017.
2. Gribble PA, Bleakley CM, Caulfield BM, Docherty CL, Fourchet F, Fong DT, Hertel J, Hiller CE, Kaminski TW, McKeon PO *et al*: **Evidence review for the 2016 International Ankle Consortium consensus statement on the prevalence, impact and long-term consequences of lateral ankle sprains**. *Br J Sports Med* 2016, **50**(24):1496-1505.
3. McGovern RP, Martin RL: **Managing ankle ligament sprains and tears: current opinion**. *Open access journal of sports medicine* 2016, **7**:33-42.
4. Feger MA, Glaviano NR, Donovan L, Hart JM, Saliba SA, Park JS, Hertel J: **Current Trends in the Management of Lateral Ankle Sprain in the United States**. *Clinical journal of sport medicine : official journal of the Canadian Academy of Sport Medicine* 2017, **27**(2):145-152.
5. Shah S, Thomas AC, Noone JM, Blanchette CM, Wikstrom EA: **Incidence and Cost of Ankle Sprains in United States Emergency Departments**. *Sports health* 2016, **8**(6):547-552.
6. Edouard P, Steffen K, Junge A, Leglise M, Soligard T, Engebretsen L: **Gymnastics injury incidence during the 2008, 2012 and 2016 Olympic Games: analysis of prospectively collected surveillance data from 963 registered gymnasts during Olympic Games**. *Br J Sports Med* 2017.
7. Doherty C, Delahunt E, Caulfield B, Hertel J, Ryan J, Bleakley C: **The incidence and prevalence of ankle sprain injury: a systematic review and meta-analysis of prospective epidemiological studies**. *Sports medicine (Auckland, NZ)* 2014, **44**(1):123-140.
8. van Rijn RM, van Os AG, Bernsen RM, Luijsterburg PA, Koes BW, Bierma-Zeinstra SM: **What is the clinical course of acute ankle sprains? A systematic literature review**. *The American journal of medicine* 2008, **121**(4):324-331.e326.
9. Larmer PJ, McNair PJ, Smythe L, Williams M: **Ankle sprains: patient perceptions of function and performance of physical tasks. A mixed methods approach**. *Disability and rehabilitation* 2011, **33**(23-24):2299-2304.
10. Bleakley CM, O'Connor SR, Tully MA, Rocke LG, Macauley DC, Bradbury I, Keegan S, McDonough SM: **Effect of accelerated rehabilitation on function after ankle sprain: randomised controlled trial**. *Bmj* 2010, **340**:c1964.
11. Doherty C, Bleakley C, Delahunt E, Holden S: **Treatment and prevention of acute and recurrent ankle sprain: an overview of systematic reviews with meta-analysis**. *British Journal of Sports Medicine* 2016.
12. Hupperets MD, Verhagen EA, Heymans MW, Bosmans JE, van Tulder MW, van Mechelen W: **Potential savings of a program to prevent ankle sprain recurrence: economic evaluation of a randomized controlled trial**. *Am J Sports Med* 2010, **38**(11):2194-2200.
13. Ardevol J, Bolibar I, Belda V, Argilaga S: **Treatment of complete rupture of the lateral ligaments of the ankle: a randomized clinical trial comparing cast immobilization with functional treatment**. *Knee surgery, sports traumatology, arthroscopy : official journal of the ESSKA* 2002, **10**(6):371-377.
14. Calatayud J, Borreani S, Colado JC, Flandez J, Page P, Andersen LL: **Exercise and ankle sprain injuries: a comprehensive review**. *The Physician and sportsmedicine* 2014, **42**(1):88-93.
15. Hupperets MD, Verhagen EA, van Mechelen W: **Effect of unsupervised home based proprioceptive training on recurrences of ankle sprain: randomised controlled trial**. *Bmj* 2009, **339**:b2684.
16. Verhagen E, Bolling C: **Protecting the health of the @hlete: how online technology may aid our common goal to prevent injury and illness in sport**. *Br J Sports Med* 2015, **49**(18):1174-1178.

17. Wong SJ, Robertson GA, Connor KL, Brady RR, Wood AM: **Smartphone apps for orthopaedic sports medicine - a smart move?** *BMC sports science, medicine & rehabilitation* 2015, **7**:23.
18. Subhi Y, Bube SH, Rolskov Bojsen S, Skou Thomsen AS, Konge L: **Expert Involvement and Adherence to Medical Evidence in Medical Mobile Phone Apps: A Systematic Review**. *JMIR mHealth and uHealth* 2015, **3**(3):e79.
19. van Mechelen DM, van Mechelen W, Verhagen EA: **Sports injury prevention in your pocket?! Prevention apps assessed against the available scientific evidence: a review**. *Br J Sports Med* 2014, **48**(11):878-882.
20. Chan AW, Tetzlaff JM, Altman DG, Laupacis A, Gotzsche PC, Krle AJK, Hrobjartsson A, Mann H, Dickersin K, Berlin JA *et al*: **SPIRIT 2013 Statement: defining standard protocol items for clinical trials**. *Revista panamericana de salud publica = Pan American journal of public health* 2015, **38**(6):506-514.
21. Bandholm T, Christensen R, Thorborg K, Treweek S, Henriksen M: **Preparing for what the reporting checklists will not tell you: the PREPARE Trial guide for planning clinical research to avoid research waste**. *British Journal of Sports Medicine* 2017.
22. Slade SC, Dionne CE, Underwood M, Buchbinder R: **Consensus on Exercise Reporting Template (CERT): Explanation and Elaboration Statement**. *Br J Sports Med* 2016.

Appendix A: Exercise program.

Reading guideline: This exercise program follows the Consensus on Exercise Reporting Template (CERT) [22].

Materials: circular rubber band, chair, table, stair step, balance board or pillow

Provider: The exercises are available in the app "InjuryMap". The program has been developed by two rheumatologists and reviewed by two physiotherapists. All experts had experience in treating ALAS patients. The exercise program was then compared and adjusted to current evidence in exercise rehabilitation for acute ankle sprains.

Delivery: It is possible to perform the exercises unsupervised at home. However, participants are free to perform the exercises anywhere they see fit. Participants are encouraged to adhere to the program by reminder notifications on their phone and by the exercise progression. The progression was designed so that participants need to "complete" a number of sessions to be able to advance to more challenging exercise. This resembles some game types and might motivate participant in completing exercise sessions. Data of exercise completion are saved in the app and used to analyze adherence. With each exercise the participants must answer pain level and difficulty. If the participants records no or low pain and low difficulty in an exercise, the app choses a progression of the exercise next time the participants begins a training session.

Description: The exercise program consists of three phases with increasing difficulty. **Phase 1** focuses on stimulating ankle mobility without provoking the acute injury. The exercises are low load with respect for end range motions. **Phase 2** focuses on increasing balance and ankle stability. The phase includes several weight bearing exercises but no impacts. **Phase 3** focuses on strength and mobility. The goal is to return participants to normal activity level. The phase includes strength exercises and jumping exercises with change of direction.

Each phase consists of several exercise categories. The categories are 1) mobility, 2) stability/balance, 3) strength and 4) stretching. Within each phase there can be more than one exercise in each category. An exercise session will consist of minimum one exercise from each category, but not necessarily all exercises in a category. Exercises can also contain a number of difficulty levels. All exercises in each category must be completed on their highest difficulty before a participant can progress to the next phase. The exercises can potential cause some discomforts or pain and participants are advised that increased swelling after training session or increased pain until next day should not be tolerated.

The specific exercises in each phase are elaborated in table 2.

Table 2: Exercise program:

Phase 1

Mobility	Stability/balance	Strength	Stretch
**Mob. 1.1: Ankle bendings.**	**Stab. 1.1 Ankle Balance**	**Strength 1.1: Sitting ankle extensions I**	**Stretch 1.1: Straight leg calf stretch**
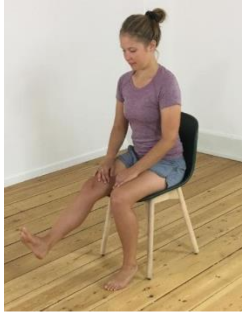	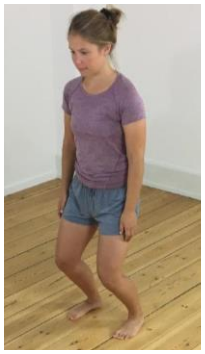	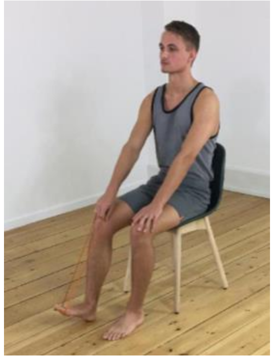	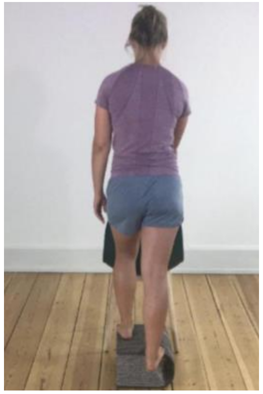
Sit on a chair. Lift the one foot from the floor. Bend the foot upwards and downwards as far as possible. If the foot is swelled the exercise can be done lying on the floor with the leg and foot raised above the heart.<br><br>10 reps each foot.	**Level I**<br>Stand with shoulder width stance and the feet pointing forward. Bend your knees as far beyond the toes as possible so that the ankles bend to their maximum. You should feel it tightens in the back of the heel and clamps in the front, but it must not be painful. Keep the tempo slow and controlled.<br><br>10 reps.	**Level I:** Sit on a chair with the knees bend. Place the rubber band under one foot just behind the toes. Hold the other end of the rubber band with one hand and tighten it. Extend the foot downwards while using the rubber band to manage resistance. Return slowly. Move as far as possible.<br><br>The rubber band needs to be so tight that it is only possible to perform	Stand on a stair step with one foot only touching the step with the forefoot and the heel free from the edge.<br>Lower the heel downwards with the knee extended until you feel a stretch in the calf muscles. Put as much weight on the leg as possible without provoking pain.<br><br>Keep stretch position 30 sec. x 3 reps.

	**Level II:** Perform the exercise while looking from side to side.	10 reps x 3 sets.<br><br>**Level II:** Perform the exercise with the knees extended.<br>15 reps x 3 sets.	
**Mob. 1.2: Ankle side tilts**	**Stab. 1.2: Calf raises I**	**Strength 1.2 Lying ankle bends**	**Stretch 1.2 Bend knee Calf stretch.**
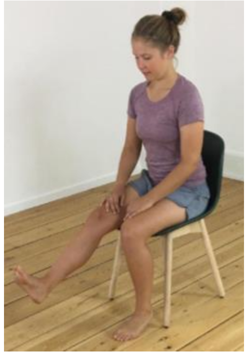	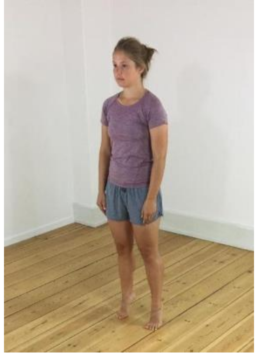	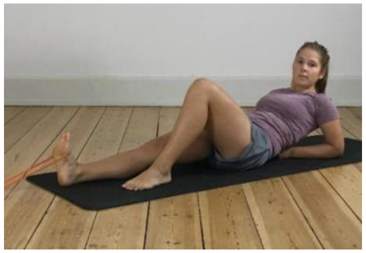	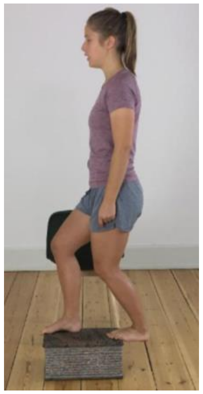
Sit on a chair. Lift the one foot from the floor. Tilt the foot outwards (eversion) and inwards (inversion) as far as possible<br><br>10 reps each foot.	**Level I:** Stand with shoulder width stance and the feet pointing forward. Raise the heels from the floor so that you’re on your tiptoes and return slowly.<br><br>10 reps.<br><br>**Level II:** Perform the exercise while looking from side to side.	Secure the rubber band on a radiator pipe or similar at ground level. Lie on your back with the injured leg extended. Place the end of the rubber band over the back of the foot just behind the toes. Bend the foot upwards while using the rubber band to manage resistance.<br><br>The rubber band needs to be so tight that it is only possible to perform 10 reps for 3 sets.	Stand on a stair step with one foot only touching the step with the forefoot and the heel free from the edge. Lower the heel downwards with the knee bended until you feel a stretch in the calf muscles. Put as much weight on the leg as possible without provoking pain.<br><br>Keep stretch position 30 sec. x 3 reps.

Mob. 1.3: Ankle circles

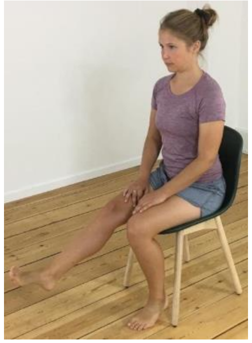

Sit on a chair. Lift the one foot from the floor. Turn the foot in large circles. Start with 5 rotations in a clockwise direction and then 5 rotations in the opposite direction.

Repeat twice

Stab. 1.3: Split stance knee bends I

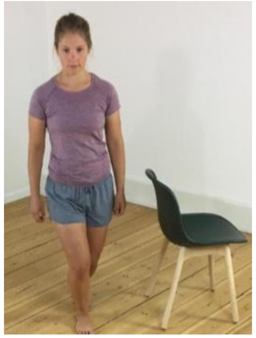

Level I: Stand with the feet in line with the injured foot in front.
Keep the balance while you slowly bend the knees beyond the toes as far as possible, so that the ankle bends to its maximum. You should feel it tightens in the back of the heel and clamps in the front, but it must not be painful.
10 repetitions.

Level II: Perform the exercise while looking from side to side.

Stretch 1.3 Hamstring stretch

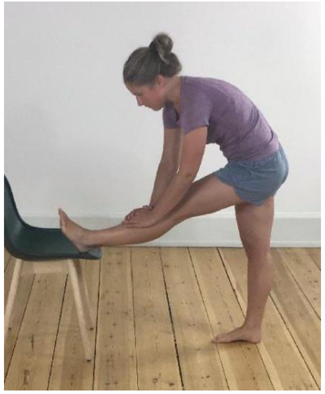

While standing towards a chair put one heel upon the seat. Lower the upper body towards the elevated leg until it stretches in the hamstrings. Be careful not to hyperextend the knees.

Keep stretch position 30 sec. x 3 reps.

Stab. 1.4
Calf raise with knee bend I

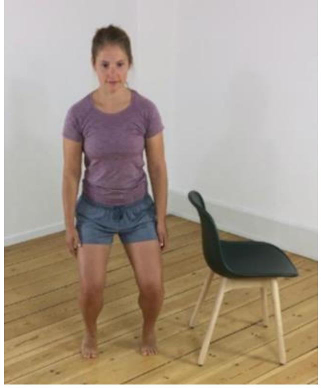

Leve II: Stand with shoulder width stance and the feet pointing forward. Bend your knees as far beyond the toes as possible so that the ankles bend to their maximum. You should feel it tightens in the back of the heel and clamps in the front, but it must not be painful. While bending your knees raise your heels so that you’re on your tiptoes and return slowly.

Repeat 5 times

Level II: Perform the exercise while looking from side to side.

Phase 2

Mobility	**Stability/balance**	**Strength**	**Stretch**
**Mob. 2.1: Ankle circles**	**Stab 2.1 One leg balance I**	**Strength 2.1: Sitting ankle inwards tilt**	**Stretch 2.1: Straight leg calf stretch**
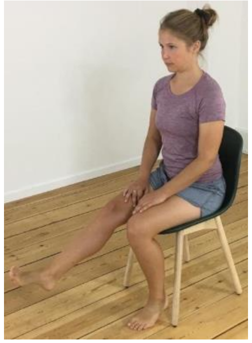	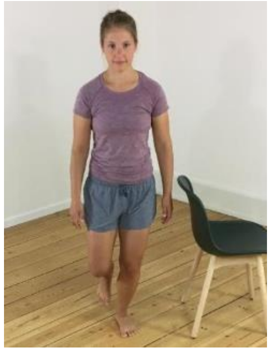	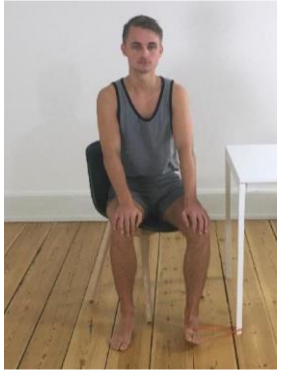	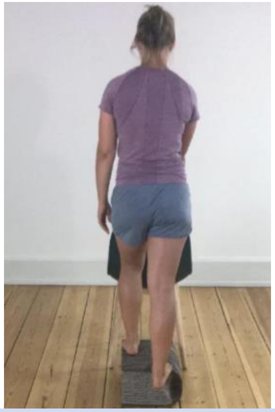
Sit on a chair. Lift the one foot from the floor. Turn the foot in large circles. Start with 5 rotations in a clockwise direction and then 5 rotations in the opposite direction.<br><br>Repeat twice	**Level I:** From a normal standing position on two legs, gradually place more weight on one leg and lift the other leg from the floor.<br>Bend the standing knee slightly and hold for 10 sec<br><br>3 reps. On each leg<br><br>**Level II:** Hold the balance for 20 sec.<br><br>**Level III:** Hold the balance for 10 seconds and look from side to side.	Sit on a chair with bended knees and a table leg beside you. Secure the rubber band on the table leg and place the other end on the forefoot just behind the toes. Slowly tilt your foot inwards and return. The heel should be kept on the floor through the movement. Tilt as much in both directions as possible. The knees and hip are kept fixed in the exercise.<br><br>The rubber band needs to be so tight that it is only possible to perform 15 reps for 3 sets. Complete the exercise with	Stand on a stair step with one foot only touching the step with the forefoot and the heel free from the edge.<br>Lower the heel downwards with the knee extended until you feel a stretch in the calf muscles. Put as much weight on the leg as possible without provoking pain.<br><br>Keep stretch position 30 sec. x 3 reps.

		both legs	
	**Stab. 2.2: Calf raise on one leg**	**Strength 2.2: Sitting ankle outwards tilt**	**Stretch 2.2 Bend knee Calf stretch.**
	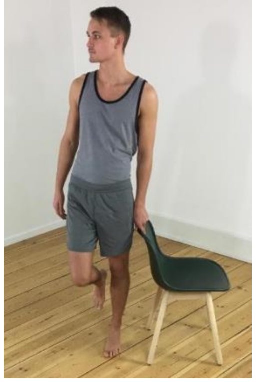	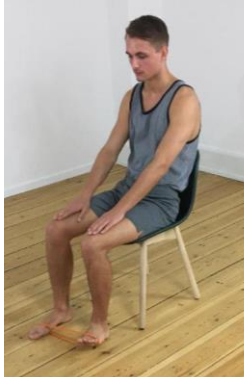	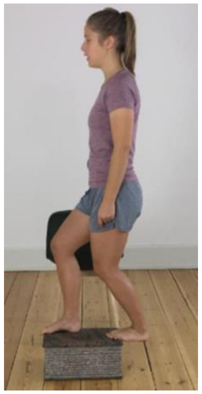
	**Level I**<br>From a normal standing position on two legs, gradually place more weight on one leg and lift the other leg.<br>While standing on one leg raise your heels so that you're on your tiptoes. Hold the balance for a couple of seconds and return slowly.<br><br>Repeat 10 times<br><br>**Level II:** Perform the exercise while looking from side to side.	Sit on a chair with bended knees. Place the rubber band around both feet just behind the toes. Keep the unscathed foot steady while you slowly tilt the injured foot outwards and return. The heel should be kept on the floor through the movement. Tilt as much in both directions as possible. The knees and hip are kept fixed in the exercise.<br><br>The rubber band needs to be so tight that it is only possible to perform 15 reps for 3 sets. Complete the exercise with both legs	Stand on a stair step with one foot only touching the step with the forefoot and the heel free from the edge.<br>Lower the heel downwards with the knee bended until you feel a stretch in the calf muscles. Put as much weight on the leg as possible without provoking pain.<br><br>Keep stretch position 30 sec. x 3 reps.

Stab. 2.3. calf raise with knee bend.

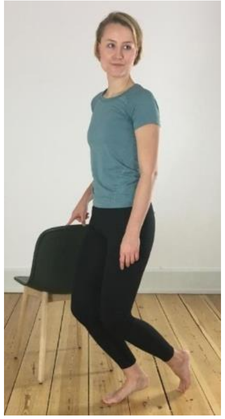

Level III: From a normal standing position on two legs, gradually place more weight on the injured leg and lift the other leg. Move the unscathed foot behind the injured and slightly put some weight on the toes for balance. From this position bend the knee beyond the toes on the injured leg. While bending raise your heel from the floor and slowly return. Complete the specified number of repetitions before switching to the other leg. To increase difficulty look from side to side while performing the repetitions.

Stretch 2.3 Hamstring stretch

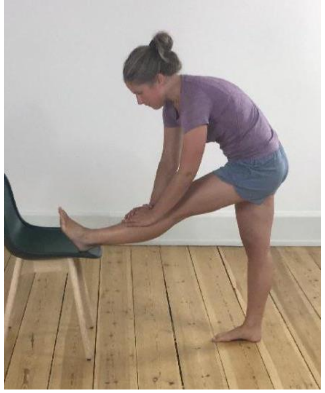

While standing towards a chair put one heel upon the seat. Lower the upper body towards the elevated leg until it stretches in the hamstrings. Be careful not to hyperextend the knees.

Keep stretch position 30 sec. x 3 reps.

5 reps on each leg

Level IV: 7 reps on each leg

Stretch 2.4 Bend knee hamstring stretch.

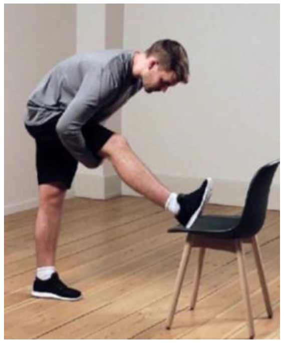

While standing towards a chair put one heel upon the seat. Bend the elevated knee slightly while lowering the upper body towards the knee until it stretches in the hamstrings. You can place your arms under the thigh for better control of the bended knee.

Keep stretch position 30 sec. x 3 reps.

Phase 3

Mobility	Stability/balance	Strength	Stretch
**Mob. 2.1: Ankle circles**	**Stab. 3.1: Balance on uneven surface**	**Strength 3.1: Sitting ankle outwards tilt**	**Stretch 3.1: Straight leg calf stretch**

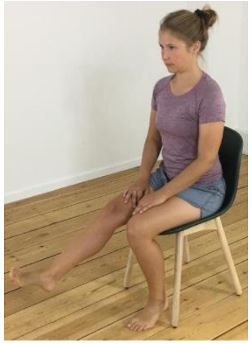

Level I: One leg

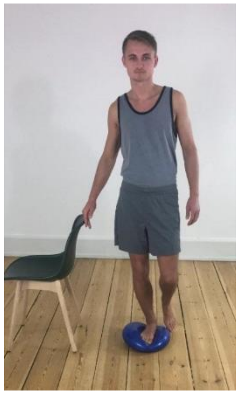

Level II: Two leg

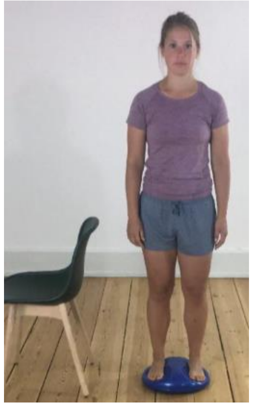

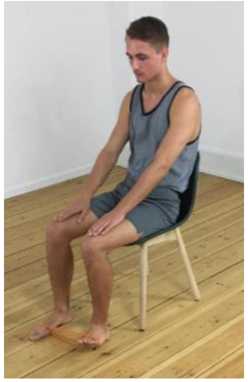

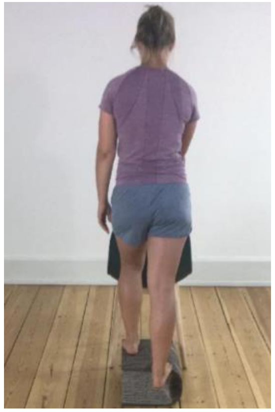

Sit on a chair. Lift the one foot from the floor. Turn the foot in large circles. Start with 5 rotations in a clockwise direction and then 5 rotations in the opposite direction.<br><br>Repeat twice	**Level I:** With a slightly bended knee stand on one leg on a balance board, hard pillow (like a couch pillow) or similar. Hold the balance without the other leg touches the ground. Repeat with the other leg.<br><br>Hold balance for 1 min. on each leg.<br><br>**Level II:** With slightly bended knees stand on both legs on a balance board, hard pillow (like a couch pillow) or similar. Slowly and with control shift your weight back on your heels and forth on your toes for 20 sec. Afterwards shift your weight from side to side for 20 sec.<br><br>Hold the balance in total for 1 min.	Sit on a chair with bended knees. Place the rubber band around both feet just behind the toes. Keep the unscathed foot steady while you slowly tilt the injured foot outwards and return. The heel should be kept on the floor through the movement. Tilt as much in both directions as possible. The knees and hip are kept fixed in the exercise.<br><br>The rubber band needs to be so tight that it is only possible to perform 15 reps for 3 sets. Complete the exercise with both legs	Stand on a stair step with one foot only touching the step with the forefoot and the heel free from the edge. Lower the heel downwards with the knee extended until you feel a stretch in the calf muscles. Put as much weight on the leg as possible without provoking pain.<br><br>Keep stretch position 30 sec. x 3 reps.

	Stab. 3.2: One leg balance III	Strength 3.2: Squat	Stretch 3.2 Bend knee Calf stretch.
	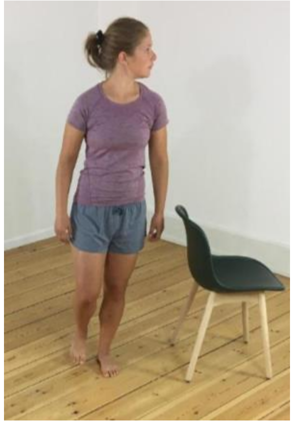	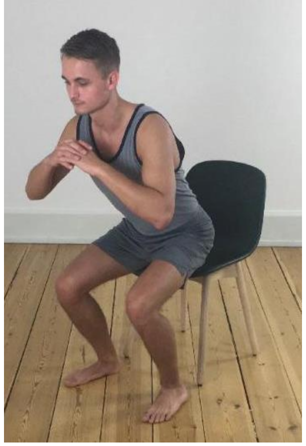	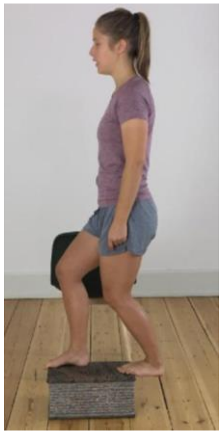
	From a normal standing position on two legs, gradually place more weight on one leg and lift the other leg from the floor.<br>Bend the standing knee slightly and hold for 10 sec while looking from side to side.<br><br>3 reps. On each leg	Place a chair a hands length behind you and stand with shoulder width stance. Weight should be equally distributed on both legs.<br>Bend your knees as would you sit down on the chair, but halt the movement just before you are sitting. Slowly return to upright position. Keep the knees oriented parallel with feet through the movement.<br><br>15 reps x 3 sets.	Stand on a stair step with one foot only touching the step with the forefoot and the heel free from the edge.<br>Lower the heel downwards with the knee bended until you feel a stretch in the calf muscles. Put as much weight on the leg as possible without provoking pain.<br><br>Keep stretch position 30 sec. x 3 reps.

	Strength 3.3: Lounges	Stretch 3.3 Hamstring stretch
	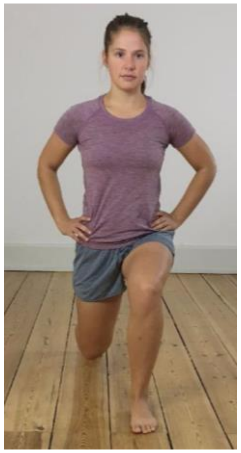	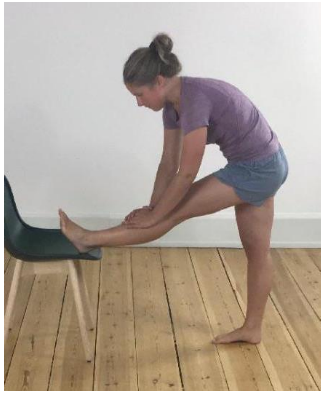
	Stand with a shoulder width stance. Make a large step forward and lower your hips until both knees are bent at about a 90 degree angle and return to upright position. Make sure your front knee is directly above your ankle and keep your upper body straight with your shoulders relaxed.<br><br>Repeat 15 times and switch leg. 3 sets for each leg.	While standing towards a chair put one heel upon the seat. Lower the upper body towards the elevated leg until it stretches in the hamstrings. Be careful not to hyperextend the knees.<br><br>Keep stretch position 30 sec. x 3 reps.

Strength 3.4: Jumps
Level I: two leg straight jump

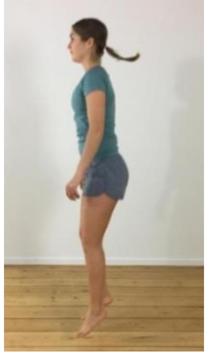

Level II: One leg straight jump

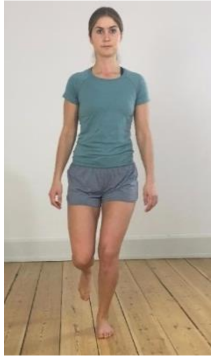

Level III: One leg forward jump

Stretch 3.4 Bend knee hamstring stretch.

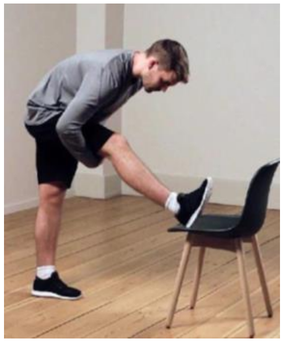

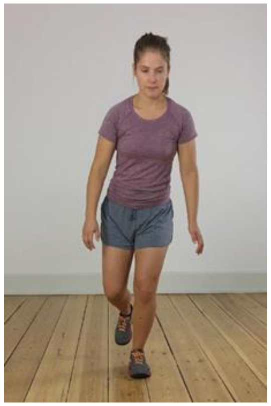

Level IV: One leg side jump

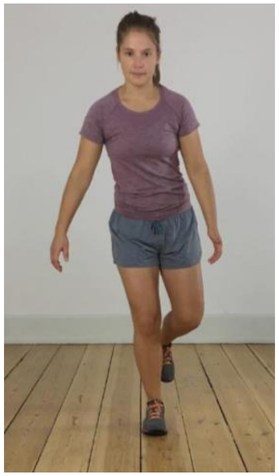

	**Level I:** two leg straight jump Stand with a shoulder width stance. Jump straight upwards and land controlled with equal weight on both legs. Make sure that the knees are oriented over the foot in the landing, so that they do not fall inwards. It should feel	While standing towards a chair put one heel upon the seat. Bend the elevated knee slightly while lowering the upper body towards the knee until it stretches in the hamstrings. You can place your arms under the thigh for better control of the bended

	even on both legs.<br><br>5 reps<br><br>**Level II:** One leg straight jump<br>Stand on one leg. Jump straight upwards and land by controlling that the knee are oriented over the foot though the landing. The knee should not fall inwards. It should feel even on both legs.<br><br>5 reps x sets on each leg.<br><br>**Level III**: One leg forward jump<br>Stand on one leg and jump back and forward. Find the balance on each landing before you jump again. It should feel even on both legs.<br><br>5 reps x 2 sets on each leg<br><br>**Level IV:** One leg side jump<br>Stand on one leg and jump from side to side. Find the balance on each landing before you jump again. It should feel even on both legs.<br><br>5 reps x sets on each leg.	knee.<br><br>Keep stretch position 30 sec. x 3 reps.

Appendix B: Outcomes.

Table 3: Outcomes.

Time	method	Outcome type
**Baseline**	Written at Emergency department.	**Participants characteristics**<br>- Name<br>- Phone number<br>- E-mail address<br>- Age<br>- Gender<br>- Weight<br>- Height<br>- Ethnicity – skin color<br>- Educational level<br>- Alcohol intake<br>- Smoking<br>**Injury**<br>- Date of injury.<br>- Injury side<br>o Left/right<br>- Dominant leg.<br>o Left/right<br>- Grade of injury.<br>I, II, III<br>- Activity when injured.<br>o Sports<br>o Work<br>o Leisure<br>- Shoe type when injured<br>o High/low heel<br>- Did you apply RICE immediate after injury<br>o Yes/no<br>- Previous ankle injury.<br>o Number total<br>o In the past six months<br>- Head injury in the past 6 month.<br>o Yes/No<br>**Work type**<br>- Which description fits the physical demands that is required for you to fulfill your job:<br>o Mostly sitting<br>o Equally sitting and walking<br>o Mostly walking

		**Sports/activity**<br>- Do you perform sports on a regular basis<br>o Yes/no<br>- How many hours a week<br>o Number of hours<br>- What type of sport do you primary perform.<br>o Indoor/court<br>o Indoor/Gymnastics<br>o Indoor/fitness<br>o Outdoor/field<br>o Outdoor track<br>o Aquatic<br>o Other (what type)
**Every week from baseline**	SMS	**Function**<br>- Are you fully weight bearing on your injured ankle<br>o If yes, Which date<br>- Can you walk naturally, without discomforts from your ankle<br>o If yes, Which date<br>**Return to activity**<br>- Are you fully back to work as before your injury<br>o If yes, which date<br>- Are you fully back to sport/activity as before your injury<br>o If yes which date<br>**Beliefs**<br>- How nervous are you for reinjury<br>o 0-10<br>- Do you feel that your ankle is stable<br>o 0-10<br>**Reinjury**<br>- how many reinjuries, have you had in the past week. If none, write "0"<br>**Harms**<br>- Have you had any discomforts when performing or in relation to the exercises.
	App	**NRS/VAS**<br>- 0-100
**Each exercise session**	App	**VAS**<br>- Before exercises<br>- After each exercise<br>**Exercise difficulty**<br>- Too hard<br>- Just right

		- Too easy
**Follow -up**	E-mail	**Willingness to pay**<br>- 10-30 kr., 31-50 kr., 51-70 kr., 70-100 kr., >100 kr.<br><br>**Satisfaction with intervention.**<br>- The result<br>o Likert scale<br>- The exercise program<br>o Likert scale<br>- The progression<br>o Likert scale<br>- User friendly<br>o Likert scale<br>- Would you recommend that the app based exercise intervention was part of normal care for Acute ankle sprains<br>o Yes/No<br>**Recovery**<br>- Do you feel that you can perform the same as before your injury.<br>o Yes/no<br>**Other treatments**<br>- Have you received treatment for your ankle sprain other than the exercises in the app<br>o Yes/no<br>- If "yes" in previous question place state which type<br>o Written explanation.
	Emergency department /App	**Feasibility**<br>Retention.<br>- How many recruited patients downloads the app<br>- How many starts the exercise program<br>- How many completes the program.<br>**Exercises/compliance:**<br>- How many exercise sessions did the participants complete<br>o Total in the intervention period.<br>o Per week in the intervention period<br>o Per week before drop out or back to activity
**Semi-structured interview.**	Telephone / meeting	**Feasibility:**<br>- Those who downloads<br>- Those who begins exercise but does not complete the program<br>- Those who complete the program<br>- The recruiting personnel.

		**Location** where exercises are performed + experiences with performing them there.

Appendix C: Participant Flow-chart

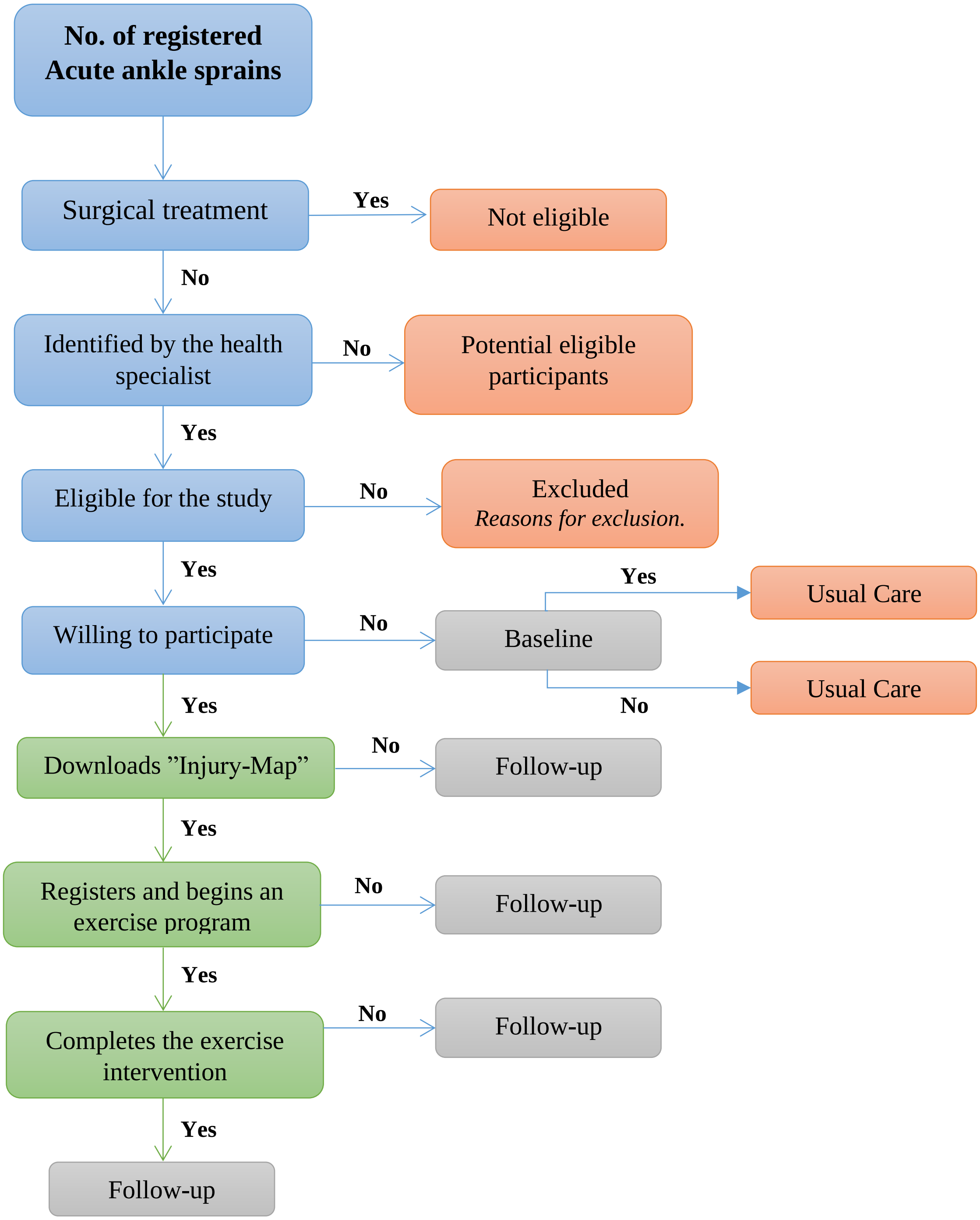

Appendix D: Patient information material

Træningstilbud til personer med akutte ankelskader.

Original titel: Evaluating the app "Injury-map" in providing exercise rehabilitation for persons with acute lateral ankle sprains – a mixed method pilot study.

Tak fordi du overvejer at deltage i dette projekt, der har til formål at forbedre behandlingen af akutte ankelskader. Din deltagelse er med til at øge viden inden for området, så fremtidig forskning og praksis kan vejledes ud fra et oplyst grundlag.

Dette projektet undersøger om en træningsapp er egnet til behandling af akutte ankelskader. Projektet forløber over 8 uger, hvor du en gang om ugen vil skulle svare på en række sms om din nuværende skade. I den 9. uge vil du modtage en e-mail med et spørgeskema angående forløbet. Du vil også kunne blive udtrukket til et telefonisk interview. Det er naturligvis frivilligt om du ønsker, at deltage i dette. Hvis du ønsker at fortsætte med træningen vil du fortsat have adgang til træningsapp'en i 3 måneder fra du registrerer dig.

Træningsapp'en kan downloades på din smartphone eller tablet. App'en vil tilpasse øvelserne til dine behov ud fra de oplysninger du giver den. Du vil blive udstyret med en træningselastik til at gennemføre programmet.

Du kan til enhver tid trække dig fra projektet. Projektdeltagelsen udelukker dig ikke fra at anvende andre behandlingsmuligheder. Dine oplysninger vil være anonyme og blive krypteret i henhold til dataloven omkring behandling af personfølsomme oplysninger. Hvis du har behov for ydereligere oplysninger er du velkommen til at tage kontakt via de informationer der står til sidst i dette dokument.

Forudsætninger for at deltage i projektet:

- Du skal have en ankelforstuvning på ydersiden af foden.
- Du skal eje en smartphone eller tablet.
- Du må ikke have pådraget dig andre skader omkring anklen eller resten af kroppen i forbindelse med ankelskaden
- Du må ikke være opereret i den pågældende ankel i forbindelse med denne skade eller tidligere.
- Du må ikke have nogen alvorlige sygdomme
- Du skal kunne læse og forstå dansk.

Hvis du beslutter dig for at deltage i projektet bedes du udfylde en samtykkeerklæring. Husk at du har ret til betænkningstid før du beslutter om du vil underskrive samtykkeerklæringen.

Hvis du ikke ønsker at deltage i træningsforløbet, vil vi stadig gerne vide noget om dig. Det vil derfor være meget prissat, hvis du vil udfylde et spørgeskema med en række oplysninger om dig selv. Du vil derefter ikke blive kontaktet yderligere og dine informationer vil blive anonymiseret.

Projektet indsamler personer indtil sommer 2018 og forventes afsluttet ultimo 2018. Projektets resultater vil som minimum blive offentliggjort.

Der forventes ingen bivirkninger i forbindelse med træningsprogrammet. Der kan dog forventes let ubehag i forbindelse med udførsel af træningsøvelser. Hvis du bliver i tvivl om en øvelse eller træningsprogrammet er hensigtsmæssig kan du til hver en tid stoppe træningen.

Projektet er initieret af Copenhagen center for Health Technology (Cashet) og InjuryMap©. Cashet er et samarbejde imellem Danmarks Tekniske Universitet (DTU), Københavns Universitet (KU) og Hvidovre Hospital. InjuryMap© er en privat ejet virksomhed med tilknytning til Copenhagen Rehab. Alle forskningsopgaver er tilknyttet PMR-C på Hvidovre hospital. InjuryMap har ansvar for de tekniske forudsætninger er til rådighed, men er ikke involveret i dataanalyse eller publicering. Projektet er finansieret af EU-fondsmidler via Institute of applied mathematics and computer science, DTU.

Jonas Bak

Klinisk Forskningscenter, Hvidovre Hospital

Jonas.bak.01@regionh.dk

Appendix E: Informed consent

The written informed consent documented used in study will the standard template S1 from The Danish National Committee on Health Research Ethics.

DET VIDENSKABSETISKE KOMITÉSYSTEM

(S1)

Informeret samtykke til deltagelse i et sundhedsvidenskabeligt forskningsprojekt.

Forskningsprojektets titel:

Erklæring fra forsøgspersonen:

Jeg har fået skriftlig og mundtlig information og jeg ved nok om formål, metode, fordele og ulemper til at sige ja til at deltage.

Jeg ved, at det er frivilligt at deltage, og at jeg altid kan trække mit samtykke tilbage uden at miste mine nuværende eller fremtidige rettigheder til behandling.

Jeg giver samtykke til, at deltage i forskningsprojektet, og har fået en kopi af dette samtykkeark samt en kopi af den skriftlige information om projektet til eget brug.

Forsøgspersonens navn: ____

Dato: _______________ Underskrift: ____

Ønsker du at blive informeret om forskningsprojektets resultat samt eventuelle konsekvenser for dig?:

Ja _____ (sæt x) Nej _____ (sæt x)

Erklæring fra den, der afgiver information:

Jeg erklærer, at forsøgspersonen har modtaget mundtlig og skriftlig information om forsøget.

Efter min overbevisning er der givet tilstrækkelig information til, at der kan træffes beslutning om deltagelse i forsøget.

Navnet på den, der har afgivet information:

Dato: _______________ Underskrift: ____
