# Supplementary material for: Using the app “Injurymap” to provide exercise rehabilitation for people with acute lateral ankle sprains seen at the Hospital Emergency Department–A mixed-method pilot study
Source: PLOS Digit Health. 2023 May 15;2(5):e0000221. doi: 10.1371/journal.pdig.0000221 (PMC10184914; doi:10.1371/journal.pdig.0000221)
Supplement: Attachment — Submitted filename: Response to reviewers.docx [file pdig.0000221.s008.docx]

PDIG-D-22-00320

Using the app “Injurymap©” to provide exercise rehabilitation for people with acute lateral ankle sprains seen at the Hospital Emergency Department – a mixed method pilot study PLOS Digital Health

Dear Dr. Bandholm,

Thank you for submitting your manuscript to PLOS Digital Health. After careful consideration, we feel that it has merit but does not fully meet PLOS Digital Health's publication criteria as it currently stands. Therefore, we invite you to submit a revised version of the manuscript that addresses the points raised during the review process.

Please submit your revised manuscript within 30 days Feb 25 2023 11:59PM. If you will need more time than this to complete your revisions, please reply to this message or contact the journal office at [digitalhealth@plos.org](mailto:digitalhealth@plos.org). When you're ready to submit your revision, log on to <https://www.editorialmanager.com/pdig/> and select the 'Submissions Needing Revision' folder to locate your manuscript file.

Please include the following items when submitting your revised manuscript:

*	A rebuttal letter that responds to each point raised by the editor and reviewer(s). You should upload this letter as a separate file labeled 'Response to Reviewers'.

*	A marked-up copy of your manuscript that highlights changes made to the original version. You should upload this as a separate file labeled 'Revised Manuscript with Track Changes'.

*	An unmarked version of your revised paper without tracked changes. You should upload this as a separate file labeled 'Manuscript'.

If you would like to make changes to your financial disclosure, please include your updated statement in your cover letter. Guidelines for resubmitting your figure files are available below the reviewer comments at the end of this letter.

We look forward to receiving your revised manuscript.

Kind regards,

Jasmit Shah

Guest Editor

PLOS Digital Health

Journal Requirements:

1. Please send a completed 'Competing Interests' statement, including any COIs declared by your co-authors. If you have no competing interests to declare, please state "The authors have declared that no competing interests exist". Otherwise please declare all competing interests beginning with the statement "I have read the journal's policy and the authors of this manuscript have the following competing interests:"

**Response:** The competing interest statement was embedded in the manuscript in the initial submission.

**Action taken:** In addition to transparently stating any COI’s in the manuscript, we have uploaded ICMJE disclosure forms for all authors as separate documents.

2. Please amend your detailed Financial Disclosure statement. This is published with the article. It must therefore be completed in full sentences and contain the exact wording you wish to be published.

a. Please clarify all sources of funding (financial or material support) for your study. List the grants (with grant number) or organizations (with url) that supported your study, including funding received from your institution.

**Response:** Our financial disclosure may not have been detailed enough in the initial submission.

**Action taken:** A detailed Financial disclosure statement has been inserted.

b. State the initials, alongside each funding source, of each author to receive each grant.

**Action taken:** Initials have been added.

c. State what role the funders took in the study. If the funders had no role in your study, please state: “The funders had no role in study design, data collection and analysis, decision to publish, or preparation of the manuscript.”

**Action taken:** The roles have been specified.

d. If any authors received a salary from any of your funders, please state which authors and which funders.

**Action taken:** Details have been added.

If you did not receive any funding for this study, please simply state: “The authors received no specific funding for this work.”

**Action taken:** Details have been added.

3. We ask that a manuscript source file is provided at Revision. Please upload your manuscript file as a .doc, .docx, .rtf or .tex.

**Action taken:** A manuscript source file has been uploaded.

4. In the online submission form, you indicated that :National legislation prohibit public sharing of a the full data set. Reviewers and others may obtain access to the data by request, and after a data processing agreement has been signed.". All PLOS journals now require all data underlying the findings described in their manuscript to be freely available to other researchers, either 1. In a public repository, 2. Within the manuscript itself, or 3. Uploaded as supplementary information.

This policy applies to all data except where public deposition would breach compliance with the protocol approved by your research ethics board. If your data cannot be made publicly available for ethical or legal reasons (e.g., public availability would compromise patient privacy), please explain your reasons by return email and your exemption request will be escalated to the editor for approval. Your exemption request will be handled independently and will not hold up the peer review process, but will need to be resolved should your manuscript be accepted for publication. One of the Editorial team will then be in touch if there are any issues.

**Action taken:** The reason has been explained and returned by email.

Please review your reference list to ensure that it is complete and correct. If you have cited papers that have been retracted, please include the rationale for doing so in the manuscript text, or remove these references and replace them with relevant current references. Any changes to the reference list should be mentioned in the rebuttal letter that accompanies your revised manuscript. If you need to cite a retracted article, indicate the article's retracted status in the References list and also include a citation and full reference for the retraction notice.

**Action taken:** The reference list has been reviewed. No changes were made.

Reviewers' comments:

Reviewer #1: In this manuscript, the authors presented excellent research that provides readers with clinically useful information that will be useful in the management of acute lateral ankle sprains. This work demonstrates the effectiveness of specific apps to provide exercise rehabilitation for people with this injury. Moreover, the research investigates important points of app use (uptake, retention, exercise adherence and satisfaction)

**Response:** Thank you for the kind words, it is greatly appreciated.

**Action taken:** None.

Reviewer #2: This paper evaluates the feasibility and primary clinical effectiveness of the Injurymap© exercise app among patients with lateral ankle sprains seen in the Emergency Department at Copenhagen University Hospital. Sixty participants were enrolled into the study, the authors found low adherence to the app, however participants were highly satisfied with the app and would recommend it to others. The authors also integrated qualitative results from semi-structured interview with a subset of the recruited participants to contextualize the engagement results. Through these interviews they identified that most participants considered ankle sprains as an innocuous injury that would recover by itself and did not feel well informed about recovery from Emergency Department healthcare professionals. The authors also identified enablers and barriers to app engagement through qualitative component. This paper was clear and succinct, written very well and reports important findings which would be relevant to the PLOS Digital Health’s readership. Please find below my comments and some minor revision recommendations:

**Response:** Thank you for the kind words, it is greatly appreciated.

**Action taken:** Please find our specific responses and actions taken below.

Introduction

-	Very clearly laid out, I really appreciated the structure, how concepts were explained, how the gaps in current research were identified and the aims were presented.

-	Found figure 1 especially useful to understand the explanatory sequential mixed method cohort study design.

-	First recommendation, it would be helpful to move “Study Design” section into the methods instead of it being in the “Introduction” section, it can be presented before “Study setting”

**Response:** We agree.

**Action taken:** The paragraph has been moved from “Introduction” to “Methods”.

Methods

-	Clearly laid out and detailed.

-	Well thought out analysis plan and explanation on why original analysis plan wasn’t completed (i.e. the shift to descriptive analyses).

-	First recommendation, in both the methods and results authors refer to participants as “people” – if possible, I would suggest call individuals who participated in the study as “participants” throughout the manuscript.

**Response:** We agree, it was an oversight in most places.

**Action taken:** “People” has been changed to “participants” in all places where we refer to people included in the study.

-	Second recommendation, on page 8 in the “material and outcome assessment” section would clarify “email” in the following sentence:

o	Participants received emails containing a link to their personal online questionnaire.

**Response:** We agree.

**Action taken:** Sentence revised from: “a browser-based software developed by Vanderbilt University. Participants received mails containing a link to their personal online questionnaire” to “a browser-based software developed by Vanderbilt University. Participants received emails containing a link to their personal online questionnaire”.

-	Third recommendation, for both the qualitative and quantitative analysis it would be helpful to report what software was used to perform the analysis.

**Response:** We agree.

**Action taken:** “Analyses were done using MS Excel, R, and MS Word” was added to the “Statistical analysis” section.

Results

-	Cleary laid out and sufficient detail is provided.

-	Found table 5 integrating the qualitative and quantitative results very informative and got the point across well.

-	First recommendation, for table Table 1 and 2, it would be helpful if a more detailed title was provided that include the sample size (e.g., Baseline characteristics of participants with LSA [n=60])

**Response:** We agree.

**Action taken:** Titles have been **revised from**: “Baseline characteristics” (table 1) and “Characteristics of the interviewed participants” (table 2) **to** “Table 1: Baseline characteristics of participants with acute lateral ankle sprains (N=60).” and “Table 2: Characteristics of the interviewed participants with acute lateral ankle sprains (N=10).”

-	Second recommendation, on page 25 for figure 5, I believe there was an error and the sentence was to say “did not” understand:

o	Patients felt stagnated when they did not understand progression in the app

**Response:** Thank you, we agree. It was an oversight. We apologize.

**Action taken: Sentence revised from:** “If participants felt, they could communicate around key concepts with the app they became frustrated.” **to** “If participants felt, they could not communicate around key concepts with the app they became frustrated.”

Discussion

-	Great discussion, overall, the authors were very transparent about their findings and did a really great job at contextualizing the results and providing thoughtful discussion about reasons for poor adherence and engagement

-	The future app optimization section was well thought out and realistic in terms of implementation.

**Response:** Thank you, we appreciate it.

Reviewer #3: This submission evaluates the use of an app to support patients with physical exercises after a LAS injury. The app is supposed to use "machine-based learning" which I take to mean machine learning, possibly to provide real-time feedback (reading on, I am still not quite clear what this refers to.) However, that is not quite clear. The app in question is InjuryMap which is not described in enough detail so that the study results can be put in context.

**Response:** Thank you for this feedback. We may not have provided enough details about the app and wrongly stated in the manuscript that it used machine learning.

**Action taken:** Paragraph **revised from**: “The exercise program for LAS consisted of three phases with increasing difficulty. Each phase consisted of four categories of exercise. The categories were 1) mobility, 2) stability/balance, 3) strength and 4) stretching. The app could adjust the difficulty for each individual exercise depending on user-feedback. A comprehensive description of the exercises can be found in Supplementary B.” **to** “The exercise program for LAS consisted of three phases with increasing difficulty. Each phase consisted of four categories of exercise. The categories were 1) mobility, 2) stability/balance, 3) strength and 4) stretching. The app could adjust the difficulty for each individual exercise depending on user-feedback. The user is asked to rate each exercise on a pain scale and difficulty scale after completing it. Their rating is used in an algorithm to calculate progression/regression for each exercise and to catch red flags. A comprehensive description of the exercises can be found in Supplementary B.”

“Machine_learning” in the abstract **revised to** “algorith-controlled progression”.

The study design is a mixed methods study without a control comparison, so it is really a usability evaluation of the app in addition to clinical outcomes. Figure 1 is confusing; it's not clear how the qual outcomes resulted from the quan: app-use and how the baseline questionnaire was applied before the intervention (which is use of the app?). It would be great to mark up Figure 1 with the number of participants that took part in the various branches. How did the distribution of the recruitment packs figure into the process?

**Response:** We agree that the figure could have some more detail to increase readability.

**Action taken:** We added numbers of participants participating in the various branches. We also added information on the numbers of participants who received the study information vs those who were enrolled.

There are several standard and validated user experience questionnaires available, why did the study not use any of them? However, the study methods are generally very good.

**Response:** When we designed the study, we were mainly focused on how the qualitative and quantitative data could mix and supplement each other in a way that would help us understand the problem better. Based on previous research, we expected that an acute ankle sprain would be considered an innocuous injury and that people with sprained ankles would stop their rehabilitation exercise a long time before full recovery. The technology – in the case the app – enabled us to objectively quantify exercise behavior during home-based rehabilitation exercise. So, our focus was more on the exercise behavior and its relation to clinical recovery than on the experience using the app. Because of this, we did not investigate specific user experience questionnaires. Arguably, the study might have benefitted from this and any app changes made could be evaluated using a standardized questionnaire. We have acknowledged this in the discussion.

**Action taken:** The app section in the Discussion was **revised from**: “With regards to the statistics in the app, we were surprised that participants did not consider them important for their motivation. It was surprising because many thought the process statistics presented after each completed session were a measure of their clinical recovery despite the fact it only reflected the program process. This is, however, similar to the finding from Liao et al. (36) who found that visual demonstration was perceived as the only important motivational factor among 52 app design features including reminder and statistical functions. A reason that the statistics are not found to be motivating could be that they are generic and not user tailored.  A patient-specific goal setting may enhance users work towards those goals (37,38) and a scale like the Patient Specific Functional Scale (39) would be easy to include as an app feature. Finally – and also related to goal setting - the app could potentially benefit from some educational information initially about what an ankle sprain is, and how much rehabilitation exercise is needed for recovery. This should also include the importance of continuing exercise when symptoms decrease so that the risk of recurrent sprains is decreased.”

**to:** “With regards to the statistics in the app, we were surprised that participants did not consider them important for their motivation. It was surprising because many thought the process statistics presented after each completed session were a measure of their clinical recovery despite the fact it only reflected the program process. This is, however, similar to the finding from Liao et al. (36) who found that visual demonstration was perceived as the only important motivational factor among 52 app design features including reminder and statistical functions. A reason that the statistics are not found to be motivating could be that they are generic and not user tailored.  A patient-specific goal setting may enhance users work towards those goals (37,38) and a scale like the Patient Specific Functional Scale (39) would be easy to include as an app feature. Finally – and also related to goal setting - the app could potentially benefit from some educational information initially about what an ankle sprain is, and how much rehabilitation exercise is needed for recovery. This should also include the importance of continuing exercise when symptoms decrease so that the risk of recurrent sprains is decreased. A standard user experience questionnaire could then be used for evaluation of any app changes made.”

In terms of results, there are some sobering figures hidden in the supplementary material that probably should be highlighted in the main submission: adherence is really low and there are 2-3 patients who do a lot of exercises, and then there is a very long tail of people not doing much. The median is 5.5 exercises over the course of 4 months! In terms of physical rehabilitation this is not enough. I think the satisfaction also needs to be taken with a pinch of salt: I think they were being nice about the feedback when they clearly didn't use the free app. It is also not a surprise that there is no reliable clinical outcome as they didn't adhere to the exercises.

**Response:** We agree that 5.5 exercise sessions over four months do not constitute a sufficient recovery stimulus. We also agree that the reported satisfaction should be taken with a grain of salt. We wanted the study to be pragmatic and to reflect clinical practice as much as possible. In clinical practice patients do not show up for appointments they made themselves or they do not perform prescribed exercise even though a clinician carefully instructed them in the program initially. They may even report that they exercised more than they did (1) likely because they do not want to disappoint the health care professional they are about to revisit. With the study, we wanted to capture a real clinical practice picture – warts and all – and report it transparently. We wanted it to be an initial look into exercise behavior now that we have technology available that can deliver rehabilitation exercise programs and objectively monitor adherence. Figure 4 holds the summary statistics for adherence and Supplementary C the individual participatory data. We have added tect to emphasized that most participants performed few or very few sessions (see below) and refer to the supplementary for data at the individual level (see below). We also state in the discussion that “If we consider 24 exercise sessions to be an acceptable adherence threshold (not considering time per session or time intervals between completed sessions) only 15% of the participants in the present study were adherent.” and added text related to taken the satisfaction with a grain of salt (see below).

(1) Rathleff MS, Bandholm T, McGirr KA, Harring SI, Sørensen AS, Thorborg K. New exercise-integrated technology can monitor the dosage and quality of exercise performed against an elastic resistance band by adolescents with patellofemoral pain: an observational study. J Physiother 201662(3): 159-163.

**Action taken:** We **revised the adherence paragraph from:** “48 participants became active users because they completed a minimum of 1 exercise session (see figure 4). The median number of completed exercise sessions in the four months period was 5.5 and ranged from 0 to 68 completed sessions (see Supplementary C). An exploratory analysis of the adherence by education level, age, work, and sports active can be found in Supplementary D.” **to** “48 participants became active users because they completed a minimum of 1 exercise session (see figure 4). The median number of completed exercise sessions in the four months period was 5.5 and ranged from 0 to 68 completed sessions with most of the participants completing few or very few sessions (see Supplementary C for completed sessions at the individual level). An exploratory analysis of the adherence by education level, age, work, and sports active can be found in Supplementary D.”

We added the following to the discussion p. 12: “The high satisfaction with the app – despite limited use – could be related to social desirability bias, that is, patients reporting what they expect would please the investigators.” and “Despite the low adherence, the participants reported high satisfaction with the app although they did not use it much.”

The discussion of the results is interesting but possibly raises even more questions than are answered. Recruitment and uptake is obviously of prime importance but it is well-known that healthcare professionals play a key role in making better choices. I was really hoping that the qualitative results would point the way to improving the app, or other apps that deliver treatment. I found it also frustrating that the discussion of the user experience didn't link back to the results.

**Response:** We agree that the user experience could have linked back better to the results and that the qualitative results could perhaps point more clearly to how the app could be improved. We have revised accordingly.

**Action taken:** The paragraph in the discussion (P.12 bottom) about LAS being an innocous injury was **changed from:** “The interviews revealed that participants in general expected a complete spontaneous recovery from the ankle sprain regardless of their actions. That people with ankle sprain believe the injury to be innocuous is anecdotally supported by several studies (1,4,10,32–34) but to our knowledge, this is the first study that has interviewed people on this perception. The perception that an ankle sprain is an innocuous injury may also be reflected in the reason for seeking medical attention at the ED, as participants primarily went to the ED because they were worried that the ankle had a fracture, not because they were worried about the consequences of a sprained – not fractured – ankle. How the perception of LAS as being an innocuous injury influences adherence to an app and an exercise program is largely unknown.”

**to:**

“The interviews revealed that participants in general expected a complete spontaneous recovery from the ankle sprain regardless of their actions. That people with ankle sprain believe the injury to be innocuous is anecdotally supported by several studies (1,4,10,32–34) but to our knowledge, this is the first study that has interviewed people on this perception. The perception that an ankle sprain is an innocuous injury may also be reflected in the reason for seeking medical attention at the ED, as participants primarily went to the ED because they were worried that the ankle had a fracture, not because they were worried about the consequences of a sprained – not fractured – ankle. How the perception of LAS as being an innocuous injury influences adherence to an app and an exercise program is largely unknown. But from the joint display of the qualitative and quantative data, it seems that the focus of their ER visit was diagnostic and that when symptoms decreased, they lost motivation for exercise. This likely resulted in low overall exercise adherence but with paridoxically high satisfaction across the different app use items.”

The section in the discussion about future app optimizing (p.14) was **changed from:** “With regards to the statistics in the app, we were surprised that participants did not consider them important for their motivation. It was surprising because many thought the process statistics presented after each completed session were a measure of their clinical recovery despite the fact it only reflected the program process. This is, however, similar to the finding from Liao et al. (36) who found that visual demonstration was perceived as the only important motivational factor among 52 app design features including reminder and statistical functions. A reason that the statistics are not found to be motivating could be that they are generic and not user tailored.  A patient-specific goal setting may enhance users work towards those goals (37,38) and a scale like the Patient Specific Functional Scale (39) would be easy to include as an app feature.”

**to**

“With regards to the statistics in the app, we were surprised that participants did not consider them important for their motivation. It was surprising because many thought the process statistics presented after each completed session were a measure of their clinical recovery despite the fact it only reflected the program process. This is, however, similar to the finding from Liao et al. (36) who found that visual demonstration was perceived as the only important motivational factor among 52 app design features including reminder and statistical functions. A reason that the statistics are not found to be motivating could be that they are generic and not user tailored.  A patient-specific goal setting may enhance users work towards those goals (37,38) and a scale like the Patient Specific Functional Scale (39) would be easy to include as an app feature. Finally – and also related to goal setting - the app could potentially benefit from some educational information initially about what an ankle sprain is, and how much rehabilitation exercise is needed for recovery. This should also include the importance of continuing exercise when symptoms decrease so that the risk of recurrent sprains is decreased.”

spelling issues:

- p. 3: an exercise session

- p. 6: is comprised of

-p. 18: questionnaire

**Response:** We apologize.

**Action taken:** Spelling issues corrected.
